# Modulation of mitochondrial dysfunction: Mechanisms and strategies for the use of natural products to treat stroke

**DOI:** 10.4103/NRR.NRR-D-25-00016

**Published:** 2025-07-05

**Authors:** Na Qin, Rujuan Liu, Rong Deng, Liuliu Shi, Lei Wang, Ting Zhu

**Affiliations:** 1 Institute of Neuroregeneration and Neurorehabilitation, Qingdao Medical College, Qingdao University, Qingdao, Shandong Province, China; 2 Department of Rehabilitation Medicine, The Affiliated Hospital of Qingdao University, Qingdao, Shandong Province, China; 3 Department of Neurosurgery, The Affiliated Hospital of Qingdao University, Qingdao, Shandong Province, China; 4 School of Traditional Chinese Pharmacy, China Pharmaceutical University, Nanjing, Jiangsu Province, China

**Keywords:** apoptosis, autophagy, hemorrhagic stroke, ischemic stroke, mitochondrial biogenesis, mitochondrial dynamics, mitochondrial dysfunction modulations, mitochondrial transport, natural products, oxidative stress

## Abstract

Modulations of mitochondrial dysfunction, which involve a series of dynamic processes such as mitochondrial biogenesis, mitochondrial fusion and fission, mitochondrial transport, mitochondrial autophagy, mitochondrial apoptosis, and oxidative stress, play an important role in the onset and progression of stroke. With a better understanding of the critical role of mitochondrial dysfunction modulations in post-stroke neurological injury, these modulations have emerged as a potential target for stroke prevention and treatment. Additionally, since effective treatments for stroke are extremely limited and natural products currently offer some outstanding advantages, we focused on the findings and mechanisms of action related to the use of natural products for targeting mitochondrial dysfunction in the treatment of stroke. Natural products achieve neuroprotective through multi-target regulation of mitochondrial dysfunction encompassing the following processes: (1) Mitochondrial biogenesis: Cordyceps and hydroxysafflor yellow A activate the peroxisome proliferator–activated receptor gamma coactivator 1-alpha/nuclear respiratory factor pathway, promote mitochondrial DNA replication and respiratory chain protein synthesis, and thereby restore energy supply in the ischemic penumbra. (2) Mitochondrial dynamics balance: Ginsenoside Rb3 promotes Opa1-mediated neural stem cell migration and diffusion for recovery of damaged brain tissue. (3) Mitochondrial autophagy: Gypenoside XVII selectively eliminates damaged mitochondria via the phosphatase and tensin homolog-induced kinase 1/Parkin pathway and blocks reactive oxygen species and the NOD-like receptor protein 3 inflammasome cascade, thereby alleviating blood–brain barrier damage. (4) Anti-apoptotic mechanisms: Ginkgolide K inhibits Bax mitochondrial translocation and downregulates caspase-3/9 activity, reducing neuronal programmed death induced by ischemia-reperfusion. (5) Oxidative stress regulation: Scutellarin exerts antioxidant properties and improves neurological function by modulating the extracellular signal-regulated kinase 5-Kruppel-like factor 2-endothelial nitric oxide synthase signaling pathway. (6) Intercellular mitochondrial transport: Neuroprotective effects of Chrysophanol are associated with accelerated mitochondrial transfer from astrocytes to neurons. Existing studies have confirmed that natural products exhibit neuroprotective effects through multidimensional interventions targeting mitochondrial dysfunction in both ischemic and hemorrhagic stroke models. However, their clinical translation still faces challenges, such as the difficulty in standardization due to component complexity, insufficient cross-regional clinical data, and the lack of long-term safety evaluations. Future research should aim to integrate new technologies, such as single-cell sequencing and organoid models, to deeply explore the mitochondria-targeting mechanisms of natural products and validate their efficacy through multicenter clinical trials, providing theoretical support and translational pathways for the development of novel anti-stroke drugs.

## Introduction

Stroke is a common, acute-onset, highly disabling, and lethal cerebrovascular disease, which mainly manifests as transient or persistent focal or generalized neurological dysfunction (Xie et al., 2024a, b; Li et al., 2025). Stroke is the second-leading cause of death worldwide and one of the major contributors to disability, and is characterized by high incidence, recurrence, disability, mortality, and economic burden (Daidone et al., 2024; Li et al., 2024). Cases of stroke have been increasingly reported in younger patients, and the incidence of stroke in young adults has increased dramatically (Ekker et al., 2018). The main risk factors for stroke include high blood pressure, diabetes, heart disease, high blood cholesterol levels, obesity, and smoking (Albertson and Sharma, 2014; Tu et al., 2023). Stroke remains a serious public health problem worldwide, necessitating continued research on its pathophysiologic mechanisms and identification of effective preventive and protective measures.

Mitochondrial dysfunction modulations (MDMs) are thought to be the central mechanism underlying a range of pathological changes in stroke. When stroke occurs, the mitochondria, which are the energy factories, bear the brunt of the severe damage caused by insufficient energy supply. Restoration of mitochondrial function restores energy metabolism, regulates dynamic homeostasis, and removes oxidative damage, among other changes. Natural products have shown substantial potential with some breakthroughs in promoting neural regeneration due to their multi-targeting ability, low toxicity, and biocompatibility. For example, curcumin plays an active role in neuronal repair and neuroprotection by modulating the nod-like receptor protein 3 (NLRP3) signaling pathway and promotes neural regeneration after ischemic stroke (Du et al., 2023). Rhodiola rosea glycosides promote autophagy for neuronal axon germination for nerve regeneration (Lai et al., 2024). This review addresses combinations of natural products with the ability to modulate mitochondrial function to provide new ideas and targets for stroke treatment. Conventional stroke treatment is focused on revascularization and pharmacological thrombolysis, but these approaches have limited long-term effects on neuroprotection and functional recovery (Ge et al., 2024; Xu et al., 2024). By treating MDMs as the core target, this review clarifies the mechanism by which natural products regulate mitochondrial function and promotes their clinical application as an adjunctive therapy or independent treatment option. The findings from this review can provide a theoretical foundation and scientific basis for the treatment of stroke by targeting mitochondria with natural products.

Stroke is primarily categorized into ischemic stroke (IS) and hemorrhagic stroke (HS) (Nukovic et al., 2023). IS accounts for approximately 80% of all cases of stroke and involves necrosis of brain tissue due to ischemia and hypoxia caused by the narrowing or blockage of blood vessels that supply oxygen and nutrients to the brain (Barthels and Das, 2020; Wang et al., 2024). IS is further divided into cerebral infarction and transient ischemic attack (TIA), of which cerebral infarction includes both cerebral thrombosis and cerebral embolism. HS, which accounts for approximately 20% of all cases of stroke, is a serious cerebrovascular condition characterized by bleeding within the brain tissue due to rupture of a cerebral blood vessel (Barthels and Das, 2020). HS mainly includes cerebral hemorrhage and subarachnoid hemorrhage (SAH) (Ohashi et al., 2023). Cerebral hemorrhage refers to the rupture and bleeding of blood vessels in the brain parenchyma, which lead to the formation of a hematoma, excluding traumatic brain hemorrhage (Hostettler et al., 2019). This type of hemorrhage is typically caused by high blood pressure. SAH involves the direct entry of blood into the subarachnoid space from the rupture of blood vessels on the brain’s surface and at its base, usually the rupture of a cerebral aneurysm (Sveinsson et al., 2011).

Cerebral thrombosis is an acute cerebrovascular disease caused by atherosclerosis, which results in slow blood flow, changes in blood composition or increased blood viscosity, and the formation of blood clots. The resultant narrowing or occlusion of the arterial lumen causes ischemia, hypoxia, softening, and necrosis of the corresponding part of the cerebral tissues and the emergence of focal neurological symptoms (Asada et al., 2020). When vascular endothelial cell dysfunction or endothelial damage occurs, collagen fibers located under the vascular endothelium activate platelet aggregation to form platelet clusters and attach to the vessel wall, and ultimately coagulate to form a block-thrombus (Furie and Furie, 2008). Unlike cerebral thrombosis, cerebral embolism is an obstruction caused by the propagation of a blood clot formed elsewhere, such as in the heart, to the brain. In addition, TIA, which is also known as a mini-stroke, is a brief episode of neurological dysfunction caused by focal cerebral or retinal ischemia (Caplan, 2006; Lewandowski, Rao and Silver, 2008). TIA is a precursor to the onset of IS and should be taken very seriously (Sonni and Thaler, 2013).

HS is a complex dynamic process, and hematoma expansion is an important pathological feature of HS. This condition consists of three distinct phases: 1) Initial hemorrhage: blood enters the brain parenchyma. 2) Hematoma expansion: hemorrhage around the hematoma leads to expansion of the volume of the hematoma. 3) Edema formation: edema appears around the hematoma (Elliott and Smith, 2010). Similar to IS, HS triggers an inflammatory response. One hour after cerebral hemorrhage, neutrophils, macrophages, and microglia are activated to damage brain tissue through the release of cytotoxic mediators, and other inflammatory mediators are upregulated in intracerebral hemorrhage (ICH); these inflammatory components act in a complex manner, exacerbating the brain damage and brain edema (Ren et al., 2020). SAH is followed by the risk of vasospasm and vasoconstriction, leading to decreased blood flow and potentially causing ischemia of the surrounding brain tissue (Naraoka et al., 2014). In conclusion, stroke consists of a range of disorders with different classifications and pathomechanisms. Understanding the different mechanisms of stroke is essential for developing targeted treatment strategies.

Overall, this review focuses on the molecular basis of the neuroprotective effects of natural products through regulation of mitochondrial biogenesis, mitochondrial dynamics, autophagy, and other pathways. This review aims to provide a comprehensive overview of the mechanisms underlying MDMs in stroke and the recent research progress on the use of natural products targeting MDMs in stroke. This review also highlights the direction for revealing the pathogenesis of stroke and the exploration of natural products for the treatment of stroke.

## Search Strategy

For this narrative review, we conducted a comprehensive search of the available reports describing the use of natural products for the treatment of stroke through mitochondrial modulation. To this end, we queried the PubMed, Genemedicine, and Web of Science databases. The searches focused on the years 2015 to 2025, and articles published from 2000 to 2025 were included. We searched the databases using the following keywords: herbal medicine, natural products, stroke, cerebral ischemia, ischemic stroke, HS, transient ischemic attack, subarachnoid hemorrhage, mitochondrial respiration, mitochondrial bioenergetics, apoptosis, mitochondrial autophagy, mitochondrial fusion and fission, mitochondrial transport, and oxidative stress. We selected English-language original scientific papers published up to January 2025 that dealt with the above topics, and aimed to include all relevant English-language original scientific papers on these topics. Initially, we identified 975 potentially relevant publications. After a thorough evaluation of abstracts and full texts, the most relevant studies were prioritized for inclusion, and 200 articles were finally selected for inclusion in the manuscript.

## Overview of Mitochondrial Dysfunction Modulations

Mitochondria are double-membrane organelles that are mainly responsible for the energy metabolism of the cell. Mitochondria are the main site of oxidative phosphorylation and synthesis of adenosine triphosphate (ATP) in the cell (Nunnari and Suomalainen, 2012; Andrieux et al., 2021). They are known as the “energy factory” of the cell, and their functional status is critical to cell health (Tan et al., 2021). In addition, mitochondria play key roles in maintaining intracellular calcium (Ca^2+^) homeostasis, apoptosis, lipid metabolism, and oxidative stress (Ng, Wai and Simonsen, 2021; Al-Suhaimi et al., 2024). Pathological states are often characterized by mitochondrial damage and resultant dysfunction. Mitochondrial dysfunction is categorized into primary and secondary mitochondrial dysfunction (Zong et al., 2024). Primary mitochondrial dysfunction is observed in genetic disorders and is characterized by damage to the mitochondrial respiratory chain as a result of defects in mitochondrial or nuclear genes, such as mitochondrial encephalomyopathy with hyperlactatemia and stroke-like episodes, Kearns-Sayre syndrome, and mitochondrial myopathy (Lu and Tarnopolsky, 2021). In contrast, secondary mitochondrial dysfunction is a disease state that results indirectly from disease-causing mutations through other key mitochondrial pathways, including mitochondrial biogenesis, mitochondrial dynamics, and the tricarboxylic acid (TCA) cycle (Baker et al., 2022). Mitochondrial dysfunction has been implicated in a variety of diseases, including metabolic disorders (Prasun, 2020; Moos et al., 2021), cardiovascular diseases (Peoples et al., 2019; Dai et al., 2023), neurodegenerative diseases (Golpich et al., 2017; Klemmensen et al., 2024), and neuromuscular diseases (Chaussenot and Paquis-Flucklinger, 2014; Miao et al., 2024). Mitochondrial dysfunction plays a key role in metabolic diseases, particularly diabetes. Moreover, hyperlipidemia has been shown to be associated with mitochondrial dysfunction, which accelerates apoptosis of retinal capillary cells and exacerbates the development of retinopathy (Kowluru et al., 2016). Mitochondrial dysfunction is also a common pathological feature in cardiovascular diseases. Mitochondrial dysfunction occurring in cardiomyocytes has been associated with diabetic cardiomyopathy, and alleviation of mitochondrial dysfunction can yield protective effects against diabetic cardiomyopathy (Guo et al., 2024). Neurodegenerative diseases are also strongly associated with mitochondrial dysfunction. Mitochondrial dysfunction, including increased levels of reactive oxygen species (ROS), abnormal Ca^2+^ homeostasis, and altered mitochondrial dynamics, has been reported to promote neurodegeneration and affect the course of Parkinson disease, Alzheimer’s disease (AD), and other related diseases (Subramaniam and Chesselet, 2013; Shoshan-Barmatz et al., 2018). In conclusion, mitochondrial dysfunction is a complex and multifaceted problem that plays a crucial role in the pathogenesis of various diseases, highlighting the need for approaches focused on enhancing mitochondrial function and maintaining mitochondrial health.

Mitochondrial dysfunction is increasingly recognized as a key factor in the pathophysiology of stroke, affecting neuronal survival and functional recovery after stroke (Zhang et al., 2023b). Under normal physiological conditions, mitochondria maintain a dynamic equilibrium through continuous fusion and division. This dynamic equilibrium is responsible for maintaining mitochondrial homeostasis and function, including energy metabolism, calcium buffering, ROS production, and apoptosis regulation, and it also influences the movement and rational distribution of mitochondria in cells (Xu et al., 2014; Buendia et al., 2016). The occurrence of stroke leads to a severe imbalance in the mitochondrial network, which triggers a series of pathological events. For example, after stroke, disruption of the blood–brain barrier (BBB), opening of the mitochondrial permeability transition pore (mPTP), and overproduction of ROS are key mechanisms leading to neuronal death (Liu et al., 2018; Wang et al., 2018). Additionally, mitochondrial dynamics, the balance between mitochondrial fission and fusion, play a crucial role in cell fate after a stroke (Huang et al., 2023a). Abnormal mitochondrial fission is associated with increased neuronal apoptosis; conversely, promotion of mitochondrial fusion may enhance cell survival (Zhang et al., 2021b; Shen et al., 2024). The relationship between stroke and mitochondrial dysfunction is multifaceted, involving biogenesis, oxidative stress, and mitochondrial dynamics (An et al., 2021). Thus, therapeutic strategies targeting mitochondrial function have the potential to improve the prognosis of stroke patients.

Accordingly, this review summarizes the knowledge of several common MDMs in stroke, including those involving mitochondrial biogenesis, mitochondrial fusion and fission, mitochondrial transport, mitochondrial autophagy, mitochondrial apoptosis, and oxidative stress, to investigate their potential relationships with stroke. Furthermore, this review attempts to summarize the roles of natural products in the treatment of stroke and the mechanisms underlying their therapeutic effects from the perspective of targeting MDMs. Hopefully, this review can provide a reference for developing more natural anti-stroke drugs for future neurotherapeutics.

## Natural Products Targeting Mitochondrial Biogenesis for the Treatment of Stroke

### Overview of mitochondrial biogenesis

Mitochondrial biogenesis refers to the self-renewal process by which cells increase the number and functionality of mitochondria, producing new mitochondria from pre-existing mitochondria (Popov, 2020; Jamwal, Blackburn and Elsworth, 2021). The regulatory mechanisms underlying mitochondrial biogenesis are complex, involving multiple transcription factors, signaling pathways, and protein interactions (Li et al., 2017a). The peroxisome proliferator-activated receptor gamma coactivator-1 alpha (PGC-1α)/nuclear respiratory factor (NRF) pathway is currently considered to be central to the regulation of mitochondrial biogenesis. PGC-1α functions as a co-transcriptional regulator that promotes mitochondrial biogenesis by activating various transcription factors (Jornayvaz and Shulman, 2010). Among these, the adenosine monophosphate (AMP)-activated kinase (AMPK)-PGC-1α axis and the sirtuin 1 (SIRT1)-PGC-1α pathway are the principal pathways governing mitochondrial biogenesis (Li et al., 2017a).

AMPK acts as a cellular energy sensor. When activation of AMPK is increased under conditions of energy deficiency or oxidative stress, AMPK can directly phosphorylate PGC-1α, which is then translocated from the cytoplasm to the nucleus to mediate mitochondrial biogenesis. AMPK can activate SIRT1 by promoting nicotinamide adenine dinucleotide (NAD^+^) production, and SIRT1 activation stimulates mitochondrial biogenesis through deacetylation of PGC-1α (Tian et al., 2019; Fan et al., 2024). Fasting has been shown to activate the SIRT1 mediation of PGC-1α deacetylation. Upon activation of PGC-1α, NRF expression is further increased, thereby regulating mitochondrial biogenesis. The importance of the PGC-1α-associated signaling pathway has been demonstrated in AD (Katsouri et al., 2011), Parkinson’s disease (Shin et al., 2011), Huntington disease (Cui et al., 2006), cardiovascular diseases, and diabetes mellitus (Besseiche et al., 2015), among other related diseases. Activation of PGC-1α by AMPK phosphorylation has been shown to reduce lipid accumulation and upregulate nuclear factor erythroid 2-related factor 2 (Nrf2) to promote mitochondrial biosynthesis and improve hyperlipidemia (Cheng et al., 2024). In addition, PGC-1α interacts with vitamin D receptor, which is also a transcriptional coactivator, and reduces Aβ deposition, thereby showing neuroprotective effects against AD (Wang et al., 2021).

### Mitochondrial biogenesis in stroke

**Figure 1:**
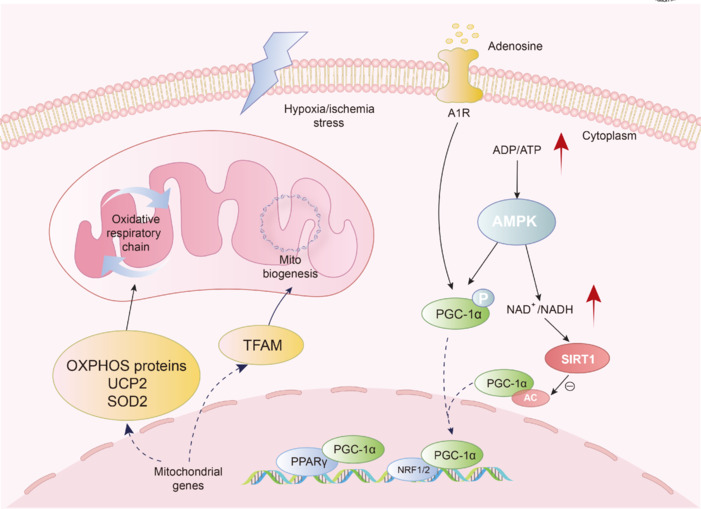
PGC-1 is essential in regulating mitochondrial biogenesis in ischemic stroke. Under ischemic and hypoxic conditions, the production of cell energy decreases, leading to an increase in intracellular ADP/ATP ratio. This change can activate the energy-sensing molecule AMPK, which phosphorylates PGC-1 and enhances its activity. In addition, AMPK activation can also activate SIRT1 by increasing the level of NAD^+^. SIRT1 deacetylates PGC-1 and activates it. Under the ischemic condition, the accelerated breakdown of cellular ATP leads to increased adenosine. Adenosine activates PGC-1 through A1R. Activated PGC-1 enters the nucleus and binds to transcription factors NRF1/2 and PPAR, which can promote the transcription of TFAM and thus promote the synthesis of mitochondrial DNA and increase the number of mitochondria. PGC-1 can also stimulate the transcription of OXPHOS protein on the electron transport chain, facilitating mitochondrial biogenesis and energy supply recovery. Red arrows indicate an increase in ratios of ADP/ATP and NAD^+^/NADH. Black solid arrows represent signal transduction processes occurring within the cytoplasm. Black dashed arrows indicate signaling pathways that involve communication between the cytoplasm and the nucleus. A1R: Adenosine A1 receptor; ADP: adenosine diphosphate; AMPK: AMP-activated protein kinase; ATP: adenosine triphosphate; IS: ischemic stroke; NRF1/2: nuclear respiratory factor 1/2; OXPHOS: oxidative phosphorylation; PGC-1α: peroxisome proliferator-activated receptor gamma coactivator 1 alpha; PPARγ: peroxisome proliferator-activated receptor gamma; SIRT1: sirtuin 1; SOD2: superoxide dismutase 2; TFAM: mitochondrial transcription factor A.

Mitochondrial biogenesis is essential for maintaining normal mitochondrial function, especially in response to energy crises or mitochondrial injury. Under stressful conditions, such as a stroke, mitochondrial biogenesis is activated to meet the cellular demand for energy. In IS, PGC-1α expression changes over time and shows a transient increase. A related study found that PGC-1α expression increased on the first day after stroke onset and decreased after the third day (Han et al., 2021). Furthermore, PGC-1α promotes autophagic flux, attenuates neuroinflammation after cerebral ischemia, and plays a key role in preventing ischemia-induced brain injury (Han et al., 2021). PGC-1α can also influence neuronal survival (Mota and Sastre, 2021). PGC-1α can attenuate the extent of hypoxia-induced neuronal damage through multiple mechanisms, such as autophagy, apoptosis, oxidative stress, and iron death (Han et al., 2022). PGC-1α overexpression promotes autophagy in neuronal cells after oxygen-glucose deprivation/reoxygenation (OGD/R), reduces ROS production, and inhibits apoptosis, thereby exerting neuroprotective effects on hippocampal neurons (Han et al., 2022). Overall, mitochondrial biogenesis plays a crucial role in cerebral ischemia and is a key factor in the process of ischemic brain injury and repair. Promoting mitochondrial biosynthesis by regulating PGC-1α plays a neuroprotective role in cerebral ischemia, and PGC-1α may be a potential target for the treatment of cerebral ischemia and related diseases (**Figure 1**).

Very few studies have described mitochondrial biogenesis in HS. One article noted that the protein level of PGC-1α, a regulator of mitochondrial biogenesis, decreases after HS in both nondiabetic and db/db mice. The mechanism of action underlying this finding involves the phosphorylation of Smad3 following HS, which leads to the downregulation of PGC-1α levels. However, the BBB-penetrable APN peptide (APNp) reverses this phenomenon and promotes the restoration of PGC-1α-related mitochondrial function (Wu et al., 2020b).

### Regulation of mitochondrial biogenesis by natural products

Cordyceps (*Cordyceps sinensis* (Berk.) Sacc., abbreviated as CS), a parasitic fungus on larval moths, is an important component of TCM formulations with a history of use spanning over a millennium. CS extracts (CSE) have been shown to reduce neurobehavioral scores and the levels of oxygen free radicals (OFR) while enhancing the concentrations of ATP, cytochrome c oxidase (COX), and mitochondrial complexes I–IV (Bai et al., 2020). The biogenesis of mitochondria is influenced by mitochondrial fusion and fission (Böckler et al., 2017). Hydroxysafflor yellow A (HSYA), a pigment derived from *Carthamus tinctorius* extract, has been shown to facilitate mitochondrial biogenesis by upregulating the expression of the fission-associated protein dynamin-1-like protein (Drp1), which is otherwise downregulated by oxygen-glucose deprivation//reoxygenation (OGD/R) and phenylalanine treatment (Chen et al., 2019). Meanwhile, the ginsenoside compound K (CK), the primary active metabolite of *Panax ginseng*, has been shown to suppress the expression of mitochondrial ubiquitin ligase 1 (Mul1), thereby modulating the ubiquitination of mitofusin 2 (Mfn2), mitochondrial dynamics, and bioenergetics. This regulation plays a crucial role in preserving mitochondrial integrity and conferring neuroprotective effects in both *in vivo* ischemia/reperfusion (I/R) and *in vitro* OGD/R models (**Additional Table 1**).

**Additional Table 1:** Natural products for the treatment of ischemic stroke by targeting mitochondrial biogenesis

Natural product	Herbal source	Biogenesis-related mode of action	*In vivo/In vitro*	Model	Optimal dose	Treatment effect	Reference
Ginsenoside compound K	*Panax ginseng*	ATP synthesis, ATP content, oxygen consumption rate, mitochondrial complex I-V, mitochondrial DNA	*In vivo In vitro*	Rat model of MCAO/R injury PC12 cell models of OGD/R injury	10 mg/kg (*in vivo*) 2, 4, and 8 μM *(in vitro)*	Ginsenoside compound K reduces the binding affinity of Mull and Mfn2 to inhibit the ubiquitination and degradation of Mfn2, thereby elevating the protein level of Mfn2 and attenuating mitochondrial translocation of Drpl.	Huang et al., 2023b
Hydroxysafflor yellow A	Carthamus tinctorius	Drp1, Opal and mitochondrial marker VDAC	*In vivo In vitro*	Rat models of MCAO/R injury Primary mouse neuron models of and PC12 cell models of OGD/R injury	5 and 20 mg/kg (*in vivo*) 1 and 10 μM (*in vitro*)	Hydroxysafflor yellow A may promote mitochondrial function and biogenesis associated with its neuroprotective effects.	Chen et al., 2019
Cordyceps sinensis extract	Cordyceps sinensis	OFR, ATP, COX, mitochondrial complexes I-IV	*In vivo*	Rat models of MCAO/R injury	1.0 g/kg	Cordyceps sinensis extract can relieve cerebral ischemia injury and exhibit protective effects via modulating the mitochondrial respiratory chain and inhibiting the mitochondrial apoptotic pathway.	Bai et al., 2020

ATP: Adenosine triphosphate; COX: cytochrome c oxidase; Drp1: dynamin-related protein; MCAO/R: middle cerebral artery occlusion reperfusion; Mfn2: mitofusin 2; OFR: oxygen free radicals; OGD/R: oxygen-glucose deprivation/re-oxygenation; Opal: optic atrophy protein 1; VDAC: voltage dependent anion channel.

## Natural Products Targeting Mitochondrial Fusion and Fission for the Treatment of Stroke

### Overview of mitochondrial fusion and fission

Mitochondria can change their form, size, and function through constant fusion and division, a physiological process known as mitochondrial dynamics (Adebayo et al., 2021; Zhou et al., 2021). The balance between mitochondrial fusion and division is critical for mitochondrial morphology, biogenesis, and quality. Mitochondrial fusion is usually a defensive reaction in which two mitochondria can enhance the resistance of the mitochondrial network by fusing their inner and outer membranes to form a single tubular mitochondrion. In contrast, mitochondrial fragmentation fragments the tubular mitochondrial network into smaller organelles, facilitating the elimination of depolarized mitochondria through mitochondrial autophagy (Gan et al., 2018). Mitochondrial fusion is mainly mediated by mitofusin 1 (Mfn1), Mfn2, and optic atrophy protein 1 (OPA1). Mitochondrial fusion is divided into outer and inner mitochondrial membrane fusion, of which outer mitochondrial membrane fusion is mediated by Mfn1 and Mfn2 located on the outer mitochondrial membrane, while inner mitochondrial membrane fusion is mediated by OPA1 (Dorn, 2013; Adebayo et al., 2021). These proteins promote fusion of mitochondrial membranes, allowing mixing of mitochondrial contents and maintaining a healthy mitochondrial network. The existing studies have demonstrated that mitochondrial fusion is caused by TM-mediated oligomerization of Mfn following guanosine triphosphate (GTP) hydrolysis (Qi et al., 2016). Mitochondrial division is primarily mediated by dynamin-related protein (Drp1).

Mitochondrial fission involves three distinct processes: (1) The cytoplasmic protein Drp1 undergoes activation through phosphorylation and subsequently associates with fission protein 1 (Fis1), mitochondrial fission factor (Mff), and the mitochondrial dynamin proteins MiD49 and MiD51. (2) Fis1, Mff, MiD49, and MiD51 direct Drp1 translocation to mitochondria. (3) In the outer mitochondrial membrane, GTP binds to Drp1 to form a tightly packed helical structure that compresses the mitochondria, thereby initiating fission and producing two separate organelles (Zhou et al., 2021). This process addresses the increasing energy needs of the cell and also maintains mitochondrial health by separating damaged mitochondria from the entire mitochondrial network. A recent study reported a novel type of division of mitochondria, in which mitochondria showed the ability to selectively separate damaged parts by tail-breaking division under the regulation of tension, as a way of controlling the quality of mitochondria (Liu et al., 2024). Kleele et al. (2021) reported two different types of mitochondrial division: a marginal division, which is mainly associated with mitochondrial autophagy, and an intermediate division, which is associated with mitochondrial proliferation. In the physiological state, mitochondrial fusion and division hold each other in check such that the mitochondria reach a form of dynamic equilibrium. Disturbances in this balance, especially when mitochondrial fusion and division are blocked, can lead to impaired mitochondrial function and ultimately result in a wide range of diseases such as AD, Parkinson’s disease, various neurological disorders, cardiovascular diseases, and cancer. In AD, inhibition of the fission pathway has been shown to alleviate mitochondrial dysfunction (Batista et al., 2021). In conclusion, mitochondrial fusion and fission are integral to cellular health, not only affecting mitochondrial morphology and function, but also playing a crucial role in disease pathogenesis and therapeutic intervention.

### Mitochondrial fusion and fission in stroke

**Figure 2:**
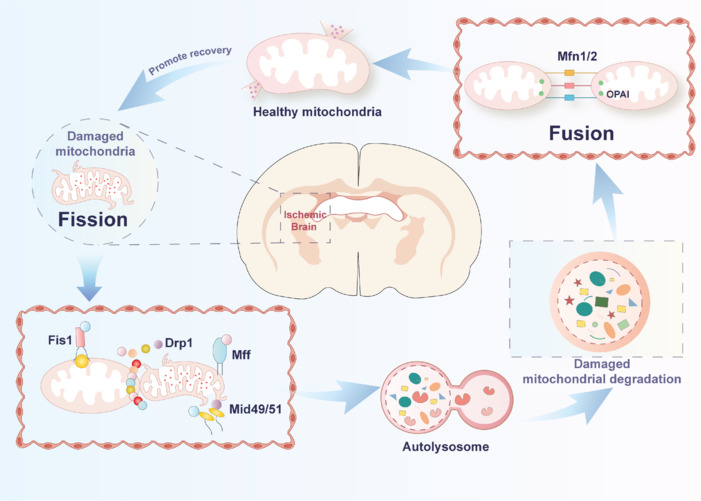
IS-induced mitochondrial dynamics and their effects on cell survival. Mitochondrial dynamics, including fusion and fission, are crucial for maintaining cellular health and responding to various stress conditions. Proteins such as Fis1, Mff, Mid49, and Mid51 interact with Drp1 to facilitate its translocation to the mitochondria, promoting mitochondrial fission. Under ischemic conditions, this process helps identify and eliminate severely damaged mitochondria by targeting them for mitophagy. Specifically, these damaged mitochondria are recognized and sequestered into autophagosomes, which then fuse with lysosomes to form autolysosomes, where degradation occurs. This degradation process reduces the negative effects of damaged mitochondria on cells. The degradation products from this process, such as amino acids and fatty acids, can be reused by the cell. For instance, amino acids can be used to synthesize new proteins such as Opa1, Mfn1, and Mfn2, which are essential for mitochondrial fusion. By recycling these components, the cell can maintain a healthy mitochondrial network and support overall cellular function. However, excessive mitochondrial fission can lead to mitochondrial dysfunction and promote cell apoptosis. Mitochondrial fusion is regulated by outer membrane fusion proteins Mfn1 and Mfn2, as well as the inner membrane fusion protein Opa1. Mitochondrial fusion plays a vital role in maintaining the structural integrity and functionality of mitochondria, reducing oxidative stress and cell apoptosis, and promoting the recovery of neurological function after a stroke. Drp1: Dynamin-related protein 1; Fis1: fission 1; IS: ischemic stroke; Mff: mitochondrial fission factor; Mfn1/2: mitochondrial fusion protein 1/2; Mid49/51: mitochondrial dynamics protein 49/51; OPA1/Opa1: optic atrophy 1.

As mentioned earlier, mitochondrial fusion and fission play important roles in maintaining mitochondrial homeostasis, and this process is particularly important in the onset, progression, and repair of damage in stroke, especially IS. IS triggers a number of changes, including mitochondrial dysfunction, which is closely related to mitochondrial fusion and division. Interestingly, mitochondrial division plays a dual role in cerebral ischemic injury (An et al, 2021). On one hand, after the occurrence of IS, which causes an imbalance in mitochondrial dynamics, the levels of fission-associated proteins increase, suggesting that mitochondrial fission begins in response to ischemic injury. Mitochondrial fission is mainly regulated by Drp1 and Fis1 and is essential for the breakdown of damaged mitochondria. The use of the mitochondrial fission inhibitor 1 (Mdivi-1) has been shown to alleviate brain damage after middle cerebral artery occlusion/reperfusion (MCAO/R) (Zuo et al., 2016). Moreover, AMPK, a key sensor of cellular energy status, recruits Drp1 to mitochondria and induces activation of mitochondrial fission, thereby alleviating mitochondrial dysfunction in response to energy stress (Toyama et al., 2016). However, excessively dividing mitochondria should be removed in time to maintain mitochondrial homeostasis. Drp1, a major factor in mitochondrial division, has been reported to have many different targets to regulate the occurrence of mitochondrial division. For example, immediately after stroke, ROS production increases. Protein kinase Cδ (PKCδ) is activated in response to oxidative stress and phosphorylates Drp1, displacing Drp1 from the cytoplasm to the mitochondria, which induces mitochondrial fragmentation and thereby leads to mitochondrial fragmentation and mitochondrial dysfunction. Thus, inhibition of PKCδ/Drp1-mediated mitochondrial division can exert a protective effect against cerebral ischemia-induced injury (Xu et al., 2017). In addition, mitochondrial division can be prevented by regulating protein kinase B (AKT), promoting its binding to Drp1, and reducing Drp1 phosphorylation levels. Unlike mitochondrial fission, mitochondrial fusion is attenuated after IS. Mitochondrial fusion promotes increased ATP production, enhances mitochondrial DNA stability, and decreases cell death (Karbowski et al., 2004). Thus, increasing mitochondrial fusion is an important way to attenuate ischemic brain injury (**Figure 2**).

The homeostasis of the mitochondrial network is critical for the maintenance of mitochondrial function and structure after HS. Excessive mitochondrial fragmentation after HS is inextricably linked to mitochondrial functional impairment. Many studies have targeted the inhibition of excessive mitochondrial division to improve mitochondrial dysfunction after HS. One study reported that excessive mitochondrial fission in perihematoma tissues of HS mice exacerbated mitochondrial ROS release and neuroinflammation. In contrast, oxytocin reduced excessive mitochondrial fission, helping to maintain the homeostasis of the mitochondrial network through the activation of p-PKA and increased phosphorylation of Drp1 at Ser637 (Yang et al., 2023). In addition, acrolein, an unsaturated aldehyde, has been shown to cause mitochondrial damage by inducing secondary brain injury after HS and is associated with increased mitochondrial Drp1 translocation and excessive mitochondrial fission. An acrolein scavenger reversed the Drp1-mediated hyperfission after HS and attenuated the morphological damage in mitochondria after HS (Wu et al., 2020a). Overall, mitochondrial fusion and division play important roles in the pathological process of HS, and by regulating their dynamic balance, brain damage can be effectively reduced and neurological recovery can be promoted.

### Regulation of mitochondrial fusion and fission by natural products

The modulation of mitochondrial homeostasis during cerebral ischemia is a critically important therapeutic strategy. CK, the principal active metabolite of *Panax ginseng*, has been shown to mitigate the mitochondrial translocation of Drp1 and counteract neuronal bioenergetic imbalances associated with I/R injury in both *in vitro* and *in vivo* models. This evidence suggests that CK may reduce the binding affinity of Mul1 and Mfn2, thereby inhibiting the ubiquitination and subsequent degradation of Mfn2. Consequently, this mechanism leads to elevated Mfn2 protein levels during cerebral I/R injury (Huang et al., 2023b). Ginsenoside Rb3, a unique saponin component found in the stems and leaves of *Panax notoginseng*, has been shown to promote Opa1-mediated mitochondrial fusion and improve mitochondrial function in both MCAO/R-induced mice and OGD/R-induced PC12 cells (Wang et al., 2025). Echinacoside significantly facilitates mitochondrial fusion progression and ameliorates cerebral injuries and behavioral deficits by upregulating Mfn2 expression in wild-type mice, but not in CK2α′^+/–^ mice (Zeng et al., 2021a). Ligustilide (LIG), the primary active component in the traditional medicinal plants *Ligusticum chuanxiong* Hort (*Chuanxiong*) and *Angelica sinensis*, mitigates IS injury by enhancing mitochondrial function. This underscores the pivotal role of LIG in regulating mitochondrial fission and mitophagy through an AMPK-dependent mechanism (Wu et al., 2022). Similarly, the alkaloid compound 2,3,5,6-tetramethylpyrazine, which is the primary bioactive constituent of *Chuanxiong*, exhibits numerous pharmacological properties, including antioxidant, anti-inflammatory, and anti-apoptotic effects. 2,3,5,6-Tetramethylpyrazine has been shown to target mitochondrial transcription factor A (TFAM) and Drp1, as well as modulate endoplasmic reticulum stress, thereby inducing neuroprotection. These findings provide an experimental basis supporting the clinical application and therapeutic potential of *Chuanxiong* in stroke treatment and underscore an alternative neuroprotective target for tetramethylpyrazine (Chang et al., 2023). Baicalin is a widely recognized natural flavonoid known for its substantial biological activities. It has been demonstrated to protect neurons by modulating mitochondrial functions through mechanisms dependent on AMPK activation (Li et al., 2017b). Ginsenoside Rg3, a tetracyclic triterpene diol saponin monomer extracted from *Panax notoginseng*, has been shown to mitigate mitochondrial oxidative stress in rats subjected to MCAO/R injury and in PC12 cells exposed to OGD/R injury. These findings robustly endorse the application of ginsenoside Rg3 as a promising therapeutic agent for I/R injury, indicating the need for further exploration of the underlying mechanisms within the Nrf2 signaling pathway (**Additional Table 2**). Pterostilbene (3′,5′-dimethoxyresveratrol) is a natural stilbene compound derived from resveratrol. It promotes mitochondrial fusion by upregulating OPA1, inhibits neuroinflammation mediated by microglia after cerebral hemorrhage, reduces lesion volume and brain edema, and mitigates BBB damage and neuronal apoptosis, thereby improving the prognosis after cerebral hemorrhage (Wu et al., 2023).

**Additional Table 2:** Natural products for the treatment of ischemic stroke by targeting mitochondrial fusion and fission

Natural product	Herbal source	Fusion and fission related mode of action	*In vivo*/I*n vitro*	Model	Optimal dose	Treatment effect	Reference
Ginsenoside compound K	*Panax ginseng*	Drpl, Fisl, Opal, Mfnl, Mfn2	*In vivo In vitro*	Rat models of MCAO/R injury PC12 cell models of OGD/R injury	10 mg/kg (*in vivo*) 2, 4, and 8 μM *(in vitro)*	Ginsenoside compound K reduces the binding affinity of Mul1 and Mfn2 to inhibit the ubiquitination and degradation of Mfn2, thereby elevating the protein level of Mfn2 and attenuating mitochondrial translocation of DRP1.	Huang et al., 2023b
Ligustilide	*Angelica sinensis* and *Ligusticum chuanxiongs*	Drpl, Fisl	*In vivo In vitro*	Rat models of MCAO/R injury HT22 cell models of OGD/R injury	20 mg/kg (*in vivo*) 5, 10, 20 μM (*in vitro*)	Ligustilide protects nerve damage against ischemic stroke by inducing Drp1-mediated mitochondrial fission via activation of AMPK signaling pathway.	Wu et al., 2022
Baicalin	*Scutellaria baicalensis Georg*	Drpl, Mfn2	*In vivo In vitro*	Rat models of MCAO/R injury PC12 cell models of OGD/R injury	100 mg/kg (*in vivo*) 0.1, 1, 10, 20 μM (*in vitro*)	Baicalin protects against hyperglycemia-aggravated I/R injury by regulating mitochondrial functions in a manner dependent on AMPK.	Li et al., 2017b
Echinacoside	*Cistanche deserticola*	Mfnl/2, Fisl, Mff, Drpl	*In vivo In vitro*	Mouse models of MCAO/R injury PC12 cell models of OGD/R injury	50 mg/kg (*in vivo*) 2.5, 5, 10 μM (*in vitro*)	Echinacoside promotes the mitochondrial fusion process by directly targeting the CK2α' subunit.	Zeng et al., 2021a
Tetramethylpyrazine	*Ligusticum wallichii Franchat*	TFAM, TOM 20, mitochondrial DNA, Drpl phosphorylation	*In vivo In vitro*	Rat models of MCAO/R injury Rat primary neuron/glia models of OGD/R injury	20 mg/kg (*in vivo*) 40 μM (*in vitro*)	Mitochondrial TFAM and Drp1 as well as endoplasmic reticulum stress could be targeted by tetramethylpyrazine to induce neuroprotection.	Chang et al., 2023
20 (R)-ginsenoside Rg3	*Panax notoginseng*	ROS, MDA, SOD, KEAPl, NRF2, HO-l	*In vivo In vitro*	Rat models of MCAO/R injury PC12 cell models of OGD/R injury	5, 10, and 20 mg/kg (*in vivo*) 5, 25, or 125 μM (*in vitro*)	20 (R)-ginsenoside Rg3 attenuates cerebral I/R injury by mitigating mitochondrial oxidative stress via the Nrf2/HO-1 signaling pathway.	Chen et al., 2024a

AMPK: AMP-activated kinase; Drpl: dynamin-related protein; Fis1: fission protein 1; HO-1: heme oxygenase-1; I/R: ischemia/reperfusion; KEAP1: kelch-like ECH-associated protein 1; Mfnl: mitofusin 1; Mfn2: mitofusin 2; Mff: mitochondrial fission factor; MCAO/R: middle cerebral artery occlusion reperfusion; Mull: mitochondrial E3 ubiquitin ligase 1; NRF2: nuclear factor erythroid 2-related factor 2; OGD/R: oxygen-glucose deprivation/reoxygenation; Opal: optic atrophy protein 1; ROS: reactive oxygen species; SOD: superoxide dismutase; TFAM: mitochondrial transcription factor A.

## Natural Products Targeting Mitochondrial Transport for the Treatment of Stroke

### Overview of mitochondrial transport

Mitochondrial transport is a key process in the maintenance of cellular function. It is especially important in neurons, where the distribution of mitochondria is critical for neuronal energy supply, calcium homeostasis, and synaptic transmission (Sheng, 2014). In neurons, mitochondrial movement patterns are categorized as stationary, retrograde, anterograde, or bidirectional. Anterograde movement, which involves movement from the neuronal cytosol to the distal part of the axon, is mediated by kinesin family proteins. In contrast, retrograde transport, which involves the movement of mitochondria from the distal end of the axon back to the cytosol, is mediated by the dynein-dynactin protein complex.

In the kinesin family of proteins, kinesin-1 (KIF5) serves as the motor protein, and the adapter protein composed of Miro-trafficking of kinesin-binding (TRAK) binds to mitochondria to drive their forward transport. KIF5, a heterotetrameric motor protein, consists of two light chains (KIF5 light chains, KLCs) and two heavy chains (KIF5 heavy chains, KHCs) (Kanai et al., 2000). The N-terminal domain of KHCs binds to microtubule proteins and hydrolyzes ATP to power mitochondrial transport; the C-terminal end is connected to the mitochondria through an adaptor protein (Sheng, 2017). In addition, KIF5 shows three isoforms: KIF5A, KIF5B, and KIF5C. KIF5A is ubiquitous, whereas KIF5B and KIF5C are specific for neurons (Sheng, 2014). The mitochondrial outer membrane receptor Miro is an atypical Ras homolog (Rashomolog, Rho) family GTPase with two Ca^2+^-binding EF-hand motifs and two GTPase structural domains (Frederick et al., 2004). Miro can bind to TRAK and work together to facilitate the attachment of motor proteins to mitochondria. Mammals have two different TRAK homologs, TRAK1 and TRAK2, while *Drosophila* carries only one TRAK gene (van Spronsen et al., 2013). TRAK1 is mainly distributed in axons and binds to KIF5 and dynein/dynactin, which is closely related to axon growth; TRAK2 is mainly distributed in dendrites and binds to dynein/dynactin, which is closely related to dendritic morphology (van Spronsen et al., 2013). This difference is mainly due to their different conformations. TRAK2 undergoes self-folding due to the interaction of the N-terminal convoluted helical region and the C-terminal structural domain, which binds to KIF5 to mutual exclusion (van Spronsen et al., 2013).

Mutations in the relevant proteins in all of the above links will result in impaired anterograde mitochondrial movement. Furthermore, in addition to the Miro-TRAK component of the articulatory proteins, syntabulin is another articulatory protein of the KIF5 motor protein that drives mitochondrial translocation into the axon and is involved in anterograde movement of mitochondria (Cai et al., 2005). Dynein-dynactin, the major motor protein driving mitochondrial retrograde movement, has been shown to use the same adaptor protein as KIF5 to connect it to mitochondria, thereby transporting mitochondria from the distal end of the axon to the cytosol (Sheng, 2017). Interestingly, syntaphilin (SNPH), another protein involved in the process of mitochondrial transport, acts as a static anchor (Kang et al., 2008). *SNPH*, as a key protein in the mitochondrial anchoring mechanism, is essential for the maintenance of normal synaptic function. Knockdown of the SNPH gene was shown to prevent normal anchoring of axonal mitochondria, which enhanced mitochondrial transport and promoted axonal regeneration after injury (Zhou et al., 2016). Thus, modulation of mitochondrial transport, or anchoring, is critical for transporting healthy mitochondria and removing damaged mitochondria to maintain synaptic function.

In neurodegenerative diseases such as AD, impairment of mitochondrial transport is associated with synaptic dysfunction and neuronal degeneration. Abnormalities in axonal mitochondrial transport induced by the accumulation of amyloid-β peptide have been identified as a factor in this impairment (Flannery and Trushina, 2019). In addition, Sheng et al. (2017) showed that enhanced mitochondrial transport can alleviate energy deficits and promote axonal regeneration, indicating that regulation of mitochondrial transport plays an important role in neuronal health and function. In addition, intercellular mitochondrial transfer is mediated by different mechanisms, which are mainly classified into three categories: (1) intercellular transfer of mitochondria via tunneling nanotubes and connexin 43 (Cx43)-mediated gap junctions, wherein tunneling nanotubes are dependent on the formation of growth-associated protein 43 (GAP43) and Cx43, which are driven by Miro1 to translocate mitochondria along the cytoskeleton (Yao et al., 2018; Tishchenko et al., 2020); (2) release of mitochondria-containing extracellular vesicles into recipient cells, which is regulated by molecules such as phosphatase and tensin homolog (PTEN)-induced putative kinase 1 (PINK1)-Parkin, CD38, and Rab7 (Crewe et al., 2021; Liang et al., 2023); (3) mitochondrial transfer dependent on the release of free mitochondria from donor cells by dividing proteins such as Drp1 and Fis1, which are captured by recipient cells through the phagocytic mechanism of acetylheparin sulfate (6-O sulfation modification) (Joshi et al., 2019; Borcherding and Brestoff, 2023). In conclusion, mitochondrial transport is a highly regulated process that is critical for neuronal function, especially in terms of energy supply and synaptic activity. Blockade of this transport mechanism has been associated with a variety of neurodegenerative diseases, highlighting the importance of tight regulation of mitochondrial transport for maintaining normal neuronal structure and function.

### Mitochondrial transport in stroke

**Figure 3:**
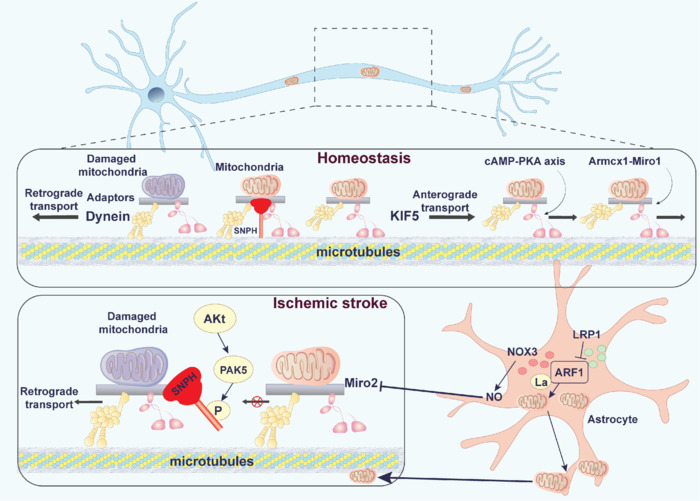
Schematic diagram illustrating mitochondrial transport during homeostasis and under ischemic stroke condition. Mitochondrial transport, which includes anterograde transport, retrograde transport, and anchoring mechanisms, is essential for maintaining the distribution and function of mitochondria within cells. Mitochondrial outer proteins Miro1 and Miro2 facilitate both anterograde and retrograde transport by linking to proteins such as Milton and TRAK1/2, connecting them to the molecular motors KIF5 and Dynein. Mitochondria are anchored and stabilized in specific cellular regions through proteins such as SNPH, ensuring a sustained energy supply to areas with high energy demand and enhancing local signal transmission. The cAMP-PKA-KIF5 and Armcx1-Miro1 signaling pathways promote mitochondrial anterograde transport and support regeneration in the central nervous system. Under ischemic conditions, reprogramming the Akt-PAK5 pathway leads to the phosphorylation of SNPH, reversing the anchoring mechanism. This process accelerates the replacement and clearance of damaged mitochondria. Astrocytes play a crucial role in mitochondrial quality control; specifically, astrocytic LRP1 promotes the transfer of healthy mitochondria to neurons and aids in recovery from ischemic stroke by inhibiting ARF1 lactylation. Furthermore, astrocytes enhance NO generation through NOX3, which modifies Miro2 via S-nitrosylation, ultimately inhibiting the retrograde transport of mitochondria. Black thick arrows (pointing right) represent anterograde transport; black thick arrows (pointing left) represent retrograde transport; black T-shaped arrow represents an inhibitory effect; black thin arrows represent molecular signaling pathways. Akt: Protein kinase B; ARF1: ADP-ribosylation factor 1; cAMP: cyclic adenosine monophosphate; IS: ischemic stroke; KIF5: kinesin family member 5; LRP1: LDL receptor related protein 1; Milton: trafficking kinesin-binding protein; NO: nitric oxide; NOX3: NADPH oxidase 3; PAK5: p21-activated kinase 5; PKA: protein kinase A; SNPH: syntaphilin; TRAK1/2: trafficking kinesin-binding protein 1/2.

In stroke, abnormalities in mitochondrial transport may lead to uneven energy distribution, neuronal dysfunction, and cell death. Maintaining normal mitochondrial transport is important for neuronal survival and functional recovery after stroke. Damaged mitochondria in neurons after I/R can be cleared by retrograde transport back to the cytosol or can be replenished with normal healthy mitochondria by anterograde movement from the cytosol to the axon terminals to restore the energy supply (Sheng, 2014). In these processes, SNPH plays a key role as a static anchor in mitochondrial transport. SNPH knockout in mice has been suggested to enhance mitochondrial transport along axons, thereby promoting axon regeneration (Zhou et al., 2016). Interestingly, the AKT-PAK5-SNPH signaling axis has been reported to regulate the switching of the SNPH static anchor, which helps remove damaged mitochondria and replenish healthy mitochondria to maintain the normal energy supply of neurons (Huang et al., 2021). Autophagy, as a major strategy for mitochondrial quality control, also plays a major role in mitochondrial axonal transport. Relevant studies have shown that I/R injury can promote the activation of autophagy, especially in the process of mitochondrial axonal transport, and the damaged mitochondria can undergo autophagy by retrograde transport back to the cytosol, which exerts neuroprotective effects. PINK1/Parkin, as one of the major pathways of autophagy, is closely related to the phosphorylation and degradation of Miro. PINK1/Parkin activates the phosphorylation of Miro to cause cessation of mitochondrial translocation (Wang et al., 2011). Mitochondrial transport occurs within individual cells and between cells. A recent study reports that astrocyte low-density lipoprotein receptor-related protein-1 (LRP1) can ameliorate stroke by regulating the malonylation modification of ADP-ribosylation factor 1 (ARF1) and facilitating the translocation of healthy mitochondria from astrocytes to damaged neurons (Zhou et al., 2024). In addition, astrocytes may also release extracellular mitochondrial particles, mediated by CD38, and enter neurons after stroke to support cell viability and recovery after stroke (Hayakawa et al., 2016). Thus, not only by regulating mitochondrial dynamics and promoting mitochondrial autophagy to facilitate intracellular mitochondrial translocation, but also by targeting astrocytes to facilitate the release of mitochondria and their translocation to neurons, new perspectives and approaches may be provided for the treatment of IS (**Figure 3**).

Maintaining normal mitochondrial function is critical for neuronal survival after HS (Diao et al., 2020), especially for regulating the distribution of healthy versus damaged mitochondria in neurons. In one study, the downregulation and overexpression of Miro1 by small interfering RNA (siRNA) and small hairpin RNA (shRNA), respectively, revealed that downregulation of Miro1 with siRNA exacerbated brain edema, increased neuronal degeneration and death in the injured region, and exacerbated neurological dysfunction after HS, while overexpression of Miro1 reversed this phenomenon and exerted neuroprotective effects. Similarly, downregulation of Miro1 by siRNA decreased both retrograde transport from axon terminals to cytosol and cis transport from cytosol to axon terminals, whereas overexpression of Miro1 enhanced mitochondrial transport, suggesting that Miro 1 overexpression reduces neuronal death after HS by promoting mitochondrial axonal transport (Li et al., 2021).

### Regulation of mitochondrial transport by natural products

Neurons can release and transfer impaired mitochondria to astrocytes, which facilitate their disposal and recycling (Davis et al., 2014). Ginsenoside Rb1 (Rb1), a predominant ginsenoside, exhibits well-documented neuroprotective effects against ischemic brain injury. Rb1 inhibits the production of ROS from the mitochondria to restrain astrocyte reactivity, and facilitates functional mitochondrial transfer to neurons. Chrysophanol, an extract from *Rheum* spp. has shown significant protective effects against cerebral I/R injury; it ameliorates brain injury by accelerating the transfer to neuronal astrocytic mitochondria and improving the morphology and function of neuronal mitochondria (Su et al., 2024). These findings suggest that astrocytes may serve as a critical strategic target for pharmacological interventions designed to safeguard neuronal integrity (Ni et al., 2022).

## Natural Products Targeting Mitochondrial Autophagy for the Treatment of Stroke

### Overview of mitochondrial autophagy

In addition to their role in cellular bioenergetics, mitochondria also regulate the levels of ROS and calcium homeostasis and are involved in the synthesis of biomolecules such as lipids, amino acids, and nucleotides (Quinn et al., 2020). Mitochondrial autophagy is a specific form of autophagy that serves as an early defense mechanism for the removal of damaged or dysfunctional mitochondria and helps prevent cellular stress and apoptosis (Pickles et al., 2018). This process is essential for maintaining the mass and functional balance of mitochondria in the cells. The process of mitochondrial autophagy involves the following steps: (1) The cell recognizes damaged or dysfunctional mitochondria on the basis of specific signals. These signals include reduced mitochondrial membrane potential, disturbed protein folding, DNA damage, or oxidative stress. (2) Subsequently, the cell will initiate autophagy, forming a double-membrane structure called an autophagosome that gradually wraps around the target mitochondria. (3) Autophagosomes fuse with lysosomes containing digestive enzymes to form autolysosomes. During this process, the encapsulated mitochondria are broken down and degraded. (4) Mitochondrial breakdown products, such as amino acids and fatty acids, are released back into the cytoplasm for cellular reuse (Lu et al., 2023). The regulation of mitochondrial autophagy involves a variety of proteins and signaling pathways. As a homeostatic regulatory mechanism *in vivo*, mitochondrial autophagy primarily occurs through two pathways: the ubiquitin-dependent pathway and the non-ubiquitin-dependent pathway. Ubiquitin-dependent pathways mainly rely on ubiquitination of mitochondrial surface proteins to promote mitochondrial autophagy. Among them, the PINK1/Parkin pathway is currently the most intensively studied pathway (Tanaka, 2020; Xie et al., 2022). Under normal conditions, guided by mitochondrial targeting sequences, PINK1 enters the inner mitochondrial membrane and is cleaved by proteases located in the mitochondrial matrix and inner membrane. Subsequently, it is released into the cytoplasm and hydrolyzed by the ubiquitin proteasome (Quinn et al., 2020). The non-ubiquitin-dependent pathway relies heavily on autophagy receptor-mediated mitochondrial autophagy. These autophagy receptors are located in the outer mitochondrial membrane and include BCL2/adenovirus E1B 19 kDa protein-interacting protein 3 (BNIP3), NIX, and FUN14 domain-containing protein 1 (FUNDC1). These receptors are able to bind directly to the microtubule-associated proteins 1A/1B light chain 3B (LC3) protein on the autophagosome membrane, thereby mediating the binding of autophagic vesicles to mitochondria. The role of mitochondrial autophagy is being increasingly recognized in a variety of diseases, including neurodegenerative diseases (Batlevi and La Spada, 2011), cardiovascular diseases (Li et al., 2022a) and cancer (Chung et al., 2020). Understanding and controlling the mechanisms of mitochondrial autophagy can provide new strategies for treating these diseases.

### Mitochondrial autophagy in stroke

**Figure 4:**
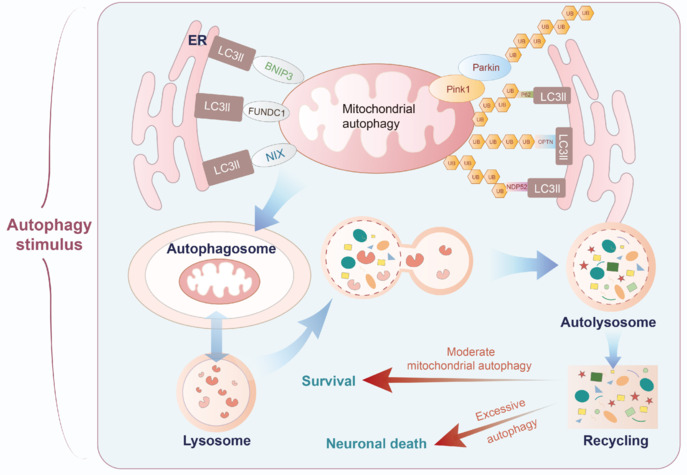
Role of mitochondrial autophagy in ischemic stroke. Under ischemic conditions, various autophagic stimuli, including AMPK, mTOR, and ULK1, play crucial roles in regulating mitochondrial autophagy. Damaged mitochondria are identified as autophagic substrates through the Pink1-Parkin pathway. When Pink1 accumulates on damaged mitochondria, it activates Parkin, which promotes the ubiquitination of these mitochondria. This process recruits autophagy adapter proteins such as p62, OPTN, and NDP52, ultimately leading to the formation of autophagosomes that are degraded by lysosomes. In addition to the Pink1-Parkin pathway, other mechanisms can initiate mitochondrial autophagy. These include NIX/BNIP3L and FUNDC1-mediated autophagy. NIX/BNIP3L can directly bind to LC3, facilitating the selective autophagy of mitochondria. Conversely, FUNDC1 enhances its binding affinity to LC3 through phosphorylation, further promoting mitochondrial autophagy. Moderate levels of autophagy protect neurons from ischemia–reperfusion injury and promote neuronal survival through the mechanisms mentioned above. However, excessive autophagy can lead to the widespread degradation of essential structural and functional proteins within cells, resulting in energy depletion and cellular damage. AMPK: AMP-activated protein kinase; ER: endoplasmic reticulum; FUNDC1: FUN14 domain containing 1; IS: ischemic stroke; LC3: microtubule-associated proteins 1A/1B light chain 3; mTOR: mechanistic target of rapamycin; NDP52: nuclear dot protein 52 kDa; NIX/BNIP3L: BCL2 interacting protein 3 like; OPTN: optineurin; Pink1: PTEN induced putative kinase 1; ULK1: Unc-51 like autophagy activating kinase 1.

The mechanisms regulating mitochondrial autophagy and its effects on neurological damage have become a focus of research on stroke. When brain tissues are ischemic and hypoxic, mitochondria become dysfunctional and PINK1 accumulates on the outer mitochondrial membrane (Furukawa et al., 2019). Concurrently, Parkin, functioning as an E3 ubiquitin ligase, is recruited to the compromised mitochondria. Parkin activation is mediated by phosphorylation in response to PINK1 signaling (Chu, 2019). Upon activation, the ubiquitin molecule is catalyzed by Parkin and translocated to a mitochondrial substrate, which is then recognized and encapsulated by autophagosomes and ultimately degraded by fusion with lysosomes. The role of mitochondrial autophagy in IS is complex. On one hand, moderate mitochondrial autophagy plays a protective role against IS, and proliferation-associated 2G4/EBP1 is ubiquitinated to induce mitochondrial autophagy via the PINK1/Parkin pathway, thereby rescuing the neuronal death induced by cerebral ischemia (Hwang et al., 2024). On the other hand, excessive mitochondrial autophagy leads to neuronal death. In neonatal mice, the absence of Atg7 after neonatal hypoxic/ischemic injury was shown to have neuroprotective effects by inhibiting mitochondrial autophagy (Koike et al., 2008). According to the relevant research reports, mesenchymal stem cells regulate autophagy through a dual function and produce neuroprotective effects against IS (He et al., 2021). The role of mitochondrial autophagy in IS can provide new perspectives and potential therapeutic targets for IS (**Figure 4**).

Mitochondrial autophagy maintains mitochondrial homeostasis by synthesizing healthy mitochondria and removing damaged mitochondria, and is essential for mitochondrial renewal after HS (Chen et al., 2024b). However, mitochondrial autophagy is a double-edged sword and plays a dual role in HS as well. On one hand, a previous study reported that activation of cholinergic anti-inflammatory pathways promoted autophagy, reduced the release of inflammatory factors, and improved neurological function after HS (Su et al., 2022). On the other hand, a recent study showed that HS-induced neuronal damage was effectively alleviated by inhibiting PTEN and, consequently, neuronal autophagy (Zhao et al., 2021). Meanwhile, Lei et al. (2022) reported that during the acute phase of HS, the HMGB1/TLR4/MyD88 axis induced neurological deficits by promoting inflammation through autophagy. Therefore, accurate regulation of autophagy is particularly important for the recovery of neurological function after HS.

### Regulation of mitochondrial autophagy by natural products

Gypenoside XVII (GP17), a distinctive constituent of *Panax notoginseng* leaf triterpenes, has demonstrated numerous pharmacological effects by modulating mitochondrial autophagy, which is linked to the interaction between the SIRT1-FOXO3A and Hif1a-BNIP3 signaling pathways (Xie et al., 2022). Similarly, CK, the principal metabolite of ginseng, suppresses autophagy-mediated apoptosis induced by OGD/R through the regulation of the AMPK-mammalian target of rapamycin (mTOR) pathway in neurons (Huang et al., 2020). Drp1-mediated mitochondrial fission facilitates mitochondrial autophagy and enhances both mitochondrial respiratory function and proteostasis in aged flies. LIG, a compound derived from the Umbelliferae family, including species such as *Chuanxiong* and *Angelica sinensis*, significantly mitigates ischemic injury by enhancing mitochondrial function. This indicates the pivotal role of LIG in modulating LIG-induced mitochondrial fission and autophagy through an AMPK-dependent pathway (Wu et al., 2022). Likewise, the active fraction of *Polyrhachis vicina* (Roger) has been shown to facilitate angiogenesis by activating mitochondrial autophagy following cerebral I/R, potentially contributing to the improvement in SIRT3-mediated regulation of the Pink1/Parkin pathway (Wei et al., 2023). *Dengzhan Xixin* injection (Yang et al., 2022b), derived from extracts of *Erigeron breviscapus* (Vaniot) Hand.-Mazz., significantly mitigates cerebral I/R injury in rats through the modulation of mitochondrial autophagy and apoptosis (**Additional Table 3**).

**Additional Table 3:** Natural products for the treatment of ischemic stroke by targeting mitochondrial autophagy

Natural product	Herbal source	Autophagy related mode of action	*In vivo/ In vitro*	Model	Optimal dose	Treatment effect	Reference
Gypenoside XVII	*Panax ginseng*	p62, ratio of LC3II/LC3I, autolysosomes, beclinl	*In vivo In vitro*	Rat models of MCAO/R injury SH-SY5Y cell models of OGD/R injury	10 and 20 mg/kg *(in vivo*) 6.25 μM *(in vitro)*	Gypenoside XVII protects against cerebral I/R injury via SIRT1-FOXO3A/Hif1a-BNIP3-mediated mitochondrial autophagy, which regulates the downstream signalling pathways of PGC-1α, SOD1, and SOD2, inhibits mitochondrial damage; alleviates oxidative stress and apoptosis.	Xie et al., 2022
Ligustilide	*Angelica sinensis* and *Ligusticum chuanxiong*	p62, ratio of LC3II/LC3I	*In vivo In vitro*	Rat models of MCAO/R injury HT22 cell models of OGD/R injury	20 mg/kg (*in vivo*) 5, 10, 20 μM *(in vitro)*	Ligustilide protects nerve damage against ischemic stroke by inducing Drpl-mediated mitochondrial fission via activation of AMPK signaling pathway.	Wu et al., 2022
Polyrhachis vicina (Roger)	---	Pinkl, Parkin, LC3-II, p62	*In vivo In vitro*	Rat models of MCAO/R injury bEnd.3 cell models of OGD/R injury	8 and 4 g raw drug/kg (*in vivo*) 0.128, 0.064, 0.032 mg/mL (*in vitro*)	AFPR upregulated SIRT3-mediated mitophagy through Pink1/Parkin signaling, accelerated damaged mitochondria clearance, and eventually improved the mitochondria function and energy production.	Wei et al., 2023
Esculetin	*Cortex Fraxini*	Bnip3, Beclinl, Pinkl, Parkin, LC-3 II/I ratio	*In vivo*	tBCCAO-treated mice	20, 80 mg/kg	ESC treatment regulated hippocampal mitophagy and mitochondrial apoptosis triggered by mitochondrial stress via the mediation of mitochondrial fragmentation during transient cerebral ischemia and reperfusion injury.	Xu et al., 2019
Compound K	*Panax ginseng C.A. Meyer*	Atg5, LC3II, Atg7	*In vitro*	OGD/R-induced PC12 cells	2, 4 and 8 μM	CK inhibits neuronal cell autophagy and apoptosis from OGD/R injury, the mechanism is due to the regulation of AMPK-mTOR pathway.	Huang et al., 2020
*Dengzhan Xixin* injection	*Erigeron breviscapus*	LC3, p62, TOM20, PINK1, Parkin	*In vivo*	Rat models of MCAO/R injury	3 and 6 mL/kg	DX exerts mitochondrial protection on MCAO-induced cerebral I/R injury of rats, which may enhance mitophagy and inhibit mitochondria-mediated apoptosis.	Yang et al., 2022b

AFPR: Polyrhachis vicina (Roger); AMPK: AMP-activated kinase; CK: compound K; DX: dengzhan xixin injection; ESC: esculetin; I/R: ischemia/reperfusion; MCAO/R: middle cerebral artery occlusion/reperfusion; OGD/R: oxygen-glucose deprivation/re-oxygenation; SIRT3: Sirtuin 3; SOD: superoxide dismutase; tBCCAO: transient bilateral common carotid artery occlusion.

## Natural Products Targeting Mitochondrial Apoptosis for the Treatment of Stroke

### Overview of mitochondrial apoptosis

The mitochondria-induced cell apoptosis pathway is primarily triggered by various physical, chemical, and biological stimuli (such as ischemia, hypoxia, Ca^2+^, nitric oxide [NO], and ROS) that increase mitochondrial outer membrane permeability. This increase leads to the release of two pro-apoptotic proteins, cytochrome c (cyt c) and apoptosis-inducing factor, from the membrane gap into the cytoplasm. Once released into the cytoplasm, cyt c binds to and activates apoptosis protease activating factor 1 (Apaf-1) and the precursor of caspase-9, forming apoptosomes that initiate downstream caspase cascade reactions, ultimately resulting in cell apoptosis. During this process, the B-cell lymphoma-2 (Bcl-2) protein family plays a crucial role in regulation. Members of the Bcl-2 family can be categorized into two groups: pro-apoptotic proteins, including Bax, Bak, and BH3-only proteins, and anti-apoptotic proteins, such as Bcl-2, Bcl-XL, and Bcl-w (Cory and Adams, 2002; Radha and Raghavan, 2017). After stimulation by apoptotic signals in the mitochondria, Bax/Bak forms oligomeric complexes that integrate into the outer mitochondrial membrane, leading to alterations in mitochondrial osmotic pressure and a reduction in transmembrane potential. This facilitates the release of cyt c from the mitochondria into the cytoplasm, thereby initiating apoptotic pathways. Additionally, apoptosis-inducing factor transfers to the nucleus, activating inactive endonucleases that cause DNA breakage, thus inducing cell apoptosis (Joza et al., 2001).

### Mitochondrial apoptosis in stroke

**Figure 5:**
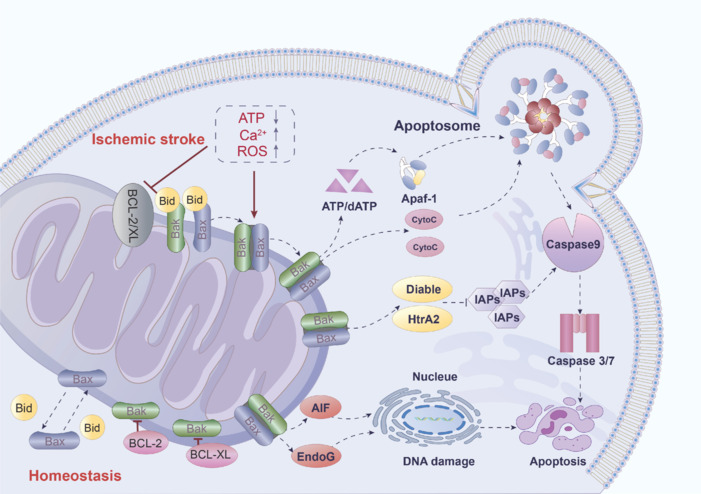
Mitochondrial apoptosis in ischemic stroke. In cases of IS, oxidative stress and elevated intracellular Ca²⁺ levels disrupt the functions of Bcl-2 and Bcl-XL, which normally provide anti-apoptotic effects. The activation of Bid initiates the activation and oligomerization of Bax and Bak, leading to increased permeability of the mitochondrial outer membrane and the release of various pro-apoptotic factors. These factors subsequently trigger the cell apoptosis signaling pathway, resulting in significant neuronal death and exacerbating brain damage. Red arrow indicates promotion; red T-shaped arrow represents inhibition; dashed arrows denote intercellular signal transduction; purple upward arrows signify upregulation; purple downward arrow represents downregulation. AIF: Apoptosis inducing factor; ATP: adenosine triphosphate; Bak: Bcl-2 antagonist/killer; Bax: bcl-2-associated X protein; Bcl-2: B-cell lymphoma 2; Bcl-XL: B-cell lymphoma-extra large; Bid: BH3 interacting-domain death agonist; Ca^2+^: calcium ion; dATP: deoxyadenosine triphosphate; HtrA2: serine protease HtrA2; IAPs: inhibitor of apoptosis protein; IS: ischemic stroke; ROS: reactive oxygen species.

The mitochondrial apoptotic pathway is one of the major pathways of apoptosis, and it plays a key role in the pathological process of stroke, especially IS. In cerebral ischemia, mitochondrial apoptosis accelerates the process of brain cell death, further exacerbating brain injury. During cerebral ischemia, due to insufficient blood supply, brain cells cannot obtain sufficient oxygen and nutrients, leading to cell dysfunction and death. One of the direct consequences of cerebral ischemia is energy metabolism disorders. Mitochondria are the main site of ATP generation, generating energy through the process of oxidative phosphorylation. When brain cells are ischemic, oxygen supply is insufficient, mitochondrial oxidative phosphorylation is blocked, and ATP production is reduced, leading to energy depletion and affecting cell survival (Guan et al., 2018). Although reperfusion after ischemia can resupply oxygen and nutrients, it can also lead to the production of large amounts of ROS, causing oxidative stress. Mitochondria are the main sources of ROS, and excessive ROS can damage mitochondria themselves, including mitochondrial DNA, proteins, and lipids, disrupting mitochondrial function and triggering mitochondria to initiate a cascade of cell apoptosis pathways by opening mPTP pores and releasing cyt c to promote cell apoptosis (Chamorro et al., 2016). Cerebral ischemia can also cause an imbalance in the intracellular and extracellular Ca^2+^ concentrations, leading to intracellular calcium overload (Ahad et al., 2020). Mitochondria absorb excess Ca^2+^ to regulate intracellular Ca^2+^ concentration, but excessive Ca^2+^ absorption can lead to mitochondrial dysfunction and even release pro-apoptotic factors such as cyt c, triggering the process of cell apoptosis. Under conditions of cerebral ischemia, these factors (energy metabolism disorders, increased oxidative stress, and calcium overload) work together on mitochondria, leading to mitochondria-induced cell apoptosis pathways. Therefore, mitochondrial apoptosis plays a crucial role in cell damage and death caused by cerebral ischemia (**Figure 5**).

Early tissue damage in the region surrounding the hematoma after cerebral hemorrhage is primarily driven by apoptosis and results from a series of injuries caused by HS. The occurrence of HS induces the opening of the mPTP and the subsequent release of cyt c into the cytoplasm, leading to the cleavage of caspase-9 (Li et al., 2024). Additionally, the ratio of Bcl-2 to Bax in brain tissue decreases after HS, further inducing apoptosis (Wang et al., 2018). A range of other protein-level changes also occur following cerebral hemorrhage, affecting apoptosis. HS is reported to be followed by mitochondrial damage and reduced mitofilin (MIC60) protein levels. Overexpression of MIC60 has been shown to reduce HS-induced neuronal death *in vivo* and *in vitro* (Deng et al., 2021). Furthermore, the reduced expression of SIRT1 in brain tissue after HS can prevent mitochondrial damage and mitochondria-dependent cell death by activating nuclear SIRT1 and promoting the deacetylation of SIRT1 via PGC-1α, thus contributing to the recovery of neurological function in HS (Zhou et al., 2018).

### Regulation of mitochondrial apoptosis by natural products

Improving mitochondrial viability, preventing membrane depolarization, and inhibiting mitochondria-dependent apoptosis cascades have become the cornerstone of IS treatment. Trametenolic acid B (TAB), which is derived from *Trametes lactinea* (Berk.) Pat., modulates molecular pathways by downregulating miR-10a mRNA and the protein expressions of Bax, cyt c, cleaved caspase-3, and cleaved caspase-9. Concurrently, TAB upregulates the protein expressions of p-PIK3CA, p-Akt, p-mTOR, Bcl-2, pro-caspase-9, and pro-caspase-3, as well as the p-PIK3CA/PIK3CA, p-Akt/Akt, and p-mTOR/mTOR ratios in rat models of cerebral I/R injury and SH-SY5Y cells subjected to OGD/R damage (Wang et al., 2019). Likewise, delavatine A, an isoquinoline alkaloid from *Incarvillea delavayi*., reduces the expression of cleaved caspase 3 and Bax, and increases Bcl-2 expression in PC12 cells exposed to OGD/R (Li et al., 2022b). Extracts from leaves of *Aglaia odorata* Lour., a well-known medicinal herb in Southeast Asia, have been shown to significantly protect PC12 cells against apoptosis by deactivating the mitochondria-dependent caspase-9/3 apoptosis pathway, potentially conferring a neuroprotective effect (Wang et al., 2020).

In addition, *Lycium barbarum* polysaccharide, a principal active component of *Lycium barbarum*, decreases the number of terminal deoxynucleotidyl transferase dUTP nick end labeling (TUNEL)-positive cells, elevates Bcl-2 protein levels, and suppresses the expression of cyt c, caspase-3, caspase-9, and cleaved poly [ADP-ribose] polymerase 1 (PARP-1) (Wang et al., 2014). Similarly, ginkgolide K markedly inhibits the activities of caspase-3 and caspase-9 in exogenous H2O2-treated PC12 cells (Ma et al., 2014). Kukoamine A is a principal bioactive constituent derived from the root bark of *Lycium (L.) chinense* Miller. It has been shown to exhibit a range of activities against oxidative stress and mitochondrial apoptosis. These activities have been confirmed through the assessment of the expression levels of Cu/Zn-superoxide dismutase (SOD), Mn-SOD, malondialdehyde (MDA), and H_2_O_2_, as well as the expression of apoptosis-related proteins such as Bax, Bcl-2, caspase-3, and cyt c (Liu et al., 2017). Moreover, Pien-Tze-Huang (PZH) is a renowned and extensively utilized TCM formula. It consists of *Panax notoginseng* (Burk.) F. H. Chen, *Calculus Bovis*, and *Moschus*, and is officially approved in China for the treatment of various inflammation-related diseases. Notably, PZH administration has been shown to significantly reduce the levels of cyt c, Bax, p53, cleaved caspase-3, and cleaved caspase-9, while concurrently increasing mitochondrial cyt c and Bcl-xl levels (Zhang et al., 2018). Additionally, PZH treatment has been shown to enhance the expression of p-AKT and p-GSK-3β (**Additional Table 4**).

**Additional Table 4:** Natural products for the treatment of ischemic stroke by targeting mitochondrial apoptosis

Natural product	Herbal source	Apoptosis-related mode of action	*In vivo/In vitro*	Model	Optimal dose	Treatment effect	Reference
Trametenolic acid B	*Trametes lactinea* (Berk.) Pat	Bax, Cyt c, cleaved-caspase-3, cleaved-caspase-9, bcl-2, pro-caspase-9, pro-caspase-3	*In vivo In vitro*	Rat models of cerebral I/R injury SH-SY5Y cell models of OGD/R injury	20, 40, and 80 mg/kg (*in vivo*) 10, 20, 40 μg/mL (*in vitro*)	Trametenolic acid B protects against ODG/R and I/R injury by suppressing miR-10a expression, activating phosphoinositide 3-kinase/AKT/mammalian target of rapamycin signaling pathway, thereby reducing mitochondrial-mediated apoptosis.	Wang et al., 2019
L-borneol	*Blumea balsamifera* (L.) DC	Cyt c, apaf-1, bad, caspase-3, bcl-2	*In vivo*	Rat models of pMCAO injury	0.05, 0.1, and 0.2 g/kg	L-borneol can achieve brain protection by downregulating the excessive expression of MCU-related signaling pathway and further inhibiting the apoptosis of neurons.	Zhang et al., 2021a
Kukoamine A	*Lycium chinense (L. chinense)* Miller	TUNEL, caspase-3, bax, bcl-2	*In vivo*	Rat models of pMCAO injury	10 mg/kg	Kukoamine A reduces infarct volume both in pre-occlusion and post-occlusion by reducing brain swelling, modulating oxidative status and inactivation mitochondrial apoptosis pathway.	Liu et al., 2017
Picroside II	*Picrorhiza*	TUNEL, Cyt c, caspase-3	*In vivo*	Rat models of cerebral I/R injury	10-20 mg/kg	Picroside II exerts a neuroprotective effect by inhibiting the mitochondria Cyt c signal pathway following ischemia/reperfusion injury in rats.	Zhang et al., 2017
Compound K	*Panax ginseng* C.A. Meyer	Bcl-2, bax, PARP, Atg5, p62, LC3II/I, Atg7	*In vitro*	Neuronal cell models of OGD/R injury	2, 4, 8 μM	Compound K inhibits neuronal cell autophagy and apoptosis from OGD/R injury. The mechanism is due to the regulation of AMPK-mTOR pathway.	Huang et al., 2020
Delavatine A	*Incarvillea delavayi*	Cleaved caspase 3, bax, bcl-2	*In vivo In vitro*	Rat models of cerebral I/R injury PC12 cell models of OGD/R injury	5, 10 mg/kg 0.5, 1.0, 2.5 μM	Delavatine A can significantly reduce apoptosis of PC12 cells caused by OGD/R, and the underlying mechanism might be related to suppressing the MKK7/JNK pathway.	Li et al., 2022b
Icariside II	*Epimedium sagittatum* Maxim	TUNEL, bax, bcl-2, cleaved caspase-3	*In vivo*	Mongolian gerbils models of bilateral common carotid arteries occlusion	20 mg/kg	Icariside II can alleviate hippocampal injury in a global cerebral I/R model, via improving microcirculatory disturbance and increasing the activity of mitochondrial complex I.	Yan et al., 2014
Cordyceps sinensis extract	*Cordyceps sinensis* (BerK.) SACC.	Bax, bcl2, Cyt c, caspase-3, caspase-8, caspase-9	*In vivo*	Rat models of MCAO	1.0 g/kg	*Cordyceps sinensis* extract can relieve cerebral ischemia injury and exhibit protective effects via modulating the mitochondrial respiratory chain and inhibiting the mitochondrial apoptotic pathway.	Bai et al., 2020
Alpinia oxyphylla Miq	---	Cyt c, cleaved caspase-3, p53/ HSP60, bcl-2/bax	*In vivo*	Rat models of MCAO injury	0.2, 0.4, 0.8 g/kg	The anti-mitochondrial apoptotic effects of the AOM extract are attributed to the interactions between upregulated p38 MAPK/p90RSK-mediated p-Bad and CREB signaling, as well as downregulated JNK/ cathepsin B-mediated Bax and p53 signaling in the peri-infarct cortex 3 days after transient MCAO.	Tsai et al., 2022
*Aglaia odorata* Lour. extract	*Aglaia odorata* Lour.	Cleaved caspase-9, cleaved caspase-3, cleaved PARP, AO/ EB staining, p53, Puma, bax, bcl-2	*In vivo In vitro*	Rat models of MCAO injury PC12 cell models of OGD/R injury	30, 100, 300 mg/kg 5, 10, 50 ng/mL	*Aglaia odorata* Lour. extract inhibits ischemic neuronal injury potentially via suppressing p53/ Puma-mediated mitochondrial apoptosis pathway.	Wang et al., 2020
Thevetiaflavone	*Wikstroemia indica*	Bcl-2, bax, caspase-3	*In vitro*	PC12 cell models of OGD/R injury	0.1, 1, 10 μM	Thevetiaflavone ameliorates injury in PC12 cells induced by OGD/R by enhancing mitochondrial function and reducing reactive oxygen species-mediated dysfunction.	Yao et al., 2017
Khat	*Catha edulis* Forssk	TUNEL	*In vivo*	Rat models of MCAO injury	3 g/kg	Khat treatment can induce apoptosis through enhancing the release of Smac/DIABLO from the mitochondria to the cytosol after transient focal ischemia which may be an important mechanism of Khat neurotoxicity.	Alsharafi et al., 2015
*Lycium barbarum* polysaccharide	*Lycium barbarum*	TUNEL, bax, bcl-2, Cyto c, caspase-3, caspase-9, cleaved PARP-1	*In vivo*	Mouse models of MCAO injury	10, 20, 40 mg/kg	The protection offered by *Lycium barbarum* polysaccharide against MCAO-induced apoptosis in cerebral ischemic mice is associated with the mitochondrial apoptosis pathway.	Wang et al., 2014
Ginkgolide K	*G. biloba* leaves	Caspase-9, caspase-8, caspase-3	*In vitro*	H_2_O_2_-induced PC12 cells	10, 50, 100 μM	Ginkgolide K protects PC12 cells from H_2_O_2_-induced apoptosis by increasing mitochondrial membrane potential, reducing reactive oxygen species levels, and ultimately inhibiting caspase-3 activity.	Ma et al., 2014
Pien-Tze-Huang	*Panax notoginseng* (Burk.) F. H. Chen, Calculus Bovis, Moschus	Cyt c, Bax, Bcl-xl, p53, caspase-3, and caspase-9	*In vivo*	Rat models of MCAO injury	90, 180, 360 mg/kg	Pien-Tze-Huang protects the brain from cerebral I/R injury *in vivo*, possibly by inhibiting mitochondria-mediated neuronal apoptosis and attenuating inflammatory responses.	Zhang et al., 2018

AOM: Alpinia oxyphylla miq; CK: compound K; CSE: cordyceps sinensis; Cyt c: cytochrome c; DA: delavatine A; I/R: ischemia/reperfusion; MCAO/R: middle cerebral artery occlusion occlusion/reperfusion; OGD/R: oxygen-glucose deprivation/re-oxygenation; pMCAO: permanent middle cerebral artery occlusion; TAB: trametenolic acid B; TUNEL: terminal deoxynucleotidyl transferase dUTP nick end labeling.

Celastrol, the main active ingredient in *Tripterygium wilfordii*, can be specifically localized in mitochondria and regulates the activation state of exchange protein directly activated by cAMP 1 by interacting with it. This process effectively hinders the binding between exchange protein directly activated by cAMP 1 and voltage-dependent anion channel 1, thereby inhibiting the opening of the mPTP, preventing the release of Ca^2+^ and cyt c from mitochondria, exerting neuroprotective effects after cerebral hemorrhage, and improving secondary brain damage induced by cerebral hemorrhage (Li et al., 2024).

Wogonin exerts a therapeutic effect on HS by binding to METTL14 and inhibiting its activity, subsequently suppressing METTL14-mediated methylation of NLRP3, which reduces pyroptosis in neuronal cells (Li et al., 2024). *Leonurus japonicus* Houtt regulates the Janus kinase (JAK)/signal transducer and activator of transcription (STAT) signaling pathway by modulating the phosphorylation of JAK1 and STAT1. Specifically, *Leonurus japonicus* inhibits the expression of the pro-apoptotic protein Bax while increasing the expression of the anti-apoptotic protein Bcl-2. This dual mechanism helps maintain neuronal survival and function, and shows significant efficacy in reducing neuronal cell death following HS (Wu et al., 2024). EGb761, an extract of *Ginkgo biloba*, significantly reduces early brain injury induced by SAH by activating the Akt signaling pathway, increasing Bcl-2 levels and reducing the expression of Bax and cleaved caspase-3. This neuroprotective effect is dose-dependent and can be abolished by the Akt inhibitor MK2206 (Yu et al., 2018). Furthermore, EGb761, by modulating the RSK1/GSK3β signaling pathway, not only reduces neuronal death in experimental cerebral hemorrhage but also promotes the formation of new blood vessels. This dual action facilitates the repair and functional recovery of brain tissue (Pan et al., 2018). Magnesium lithospermate B (MLB), a bioactive component extracted from *Salvia miltiorrhiza*, exerts notable neuroprotective effects by reducing the expression of pro-apoptotic protein cleaved caspase-3 and inflammatory cytokines. This leads to the inhibition of apoptosis and neuroinflammation, resulting in marked improvements in brain edema and neurological deficits following SAH. These neuroprotective effects are mediated through regulation of the SIRT1/nuclear factor (NF)-κB signaling pathway (Peng et al., 2018). Z-ligustilide (LIG), the primary lipophilic component derived from the traditional Chinese herb *Angelica sinensis*, has shown significant neuroprotective effects by reducing cell apoptosis. Specifically, LIG decreases the expression of pro-apoptotic proteins such as p53 and cleaved caspase-3, leading to improvements in mortality rates, neurological behavior, and vascular function in experimental models of SAH (Chen et al., 2011). High concentrations of resveratrol, by activating the phosphoinositide 3-kinase (PI3K)/Akt signaling pathway, demonstrate significant anti-apoptotic effects in EBI following experimental SAH (Zhou et al., 2014).

## Natural Products Targeting Mitochondrial Oxidative Stress for the Treatment of Stroke

### Overview of mitochondrial oxidative stress

Mitochondria are critical organelles within cells that are primarily responsible for energy metabolism. Within the mitochondrial respiratory chain, electron leakage from the electron transport chain can lead to the reduction of oxygen molecules, resulting in the formation of ROS. These ROS include superoxide anions (O_2_·^–^), H_2_O_2_, and hydroxyl radicals (·OH) (Angelova and Abramov, 2016). The generation of ROS exerts a dual effect on cellular function: while excessive levels can be detrimental, moderate concentrations serve as signaling molecules that facilitate various physiological processes, including cell proliferation, differentiation, and apoptosis (Angelova and Abramov, 2016). However, excessive ROS can lead to oxidative stress in cells, damaging cell membranes, proteins, and DNA. This damage is associated with various diseases, including cardiovascular diseases, diabetes, cancer, and particularly IS (Luo et al., 2014; Chio and Tuveson, 2017; Sabbaghziarani et al., 2024).

Cells possess an endogenous antioxidant system to counteract the potential damage caused by ROS. This system includes various antioxidant enzymes and non-enzymatic systems that can clear ROS or alleviate the damage caused by ROS. Antioxidant enzymes, including SOD, catalase, and glutathione peroxidase (GPx), can directly clear ROS or participate in the metabolic process of ROS. Non-enzymatic antioxidants, such as vitamin C, vitamin E, and glutathione (GSH), can provide electrons or hydrogen atoms to stabilize ROS, thereby reducing cell damage (Birben et al., 2012). ROS generation represents a cascade of reactions starting with the production of O_2_^–^, which can be further converted to H_2_O_2_ via SOD. The produced H_2_O_2_ is decomposed to H_2_O and O_2_ by catalase and GPx. Moreover, GPX utilizes GSH, which is oxidized to GSSG and recovered by glutathione reductase (Birben et al., 2012). Nrf2 has been demonstrated to be an essential transcription factor regulating oxidative stress and ameliorating brain damage. In response to increased oxidative stress, Nrf2 is released from kelch-like ECH-associated protein 1 (keap1) and translocated into the nucleus, where it induces the expression of antioxidant proteins, including heme oxygenase-1 (HO-1), NAD (P)H quinone oxidoreductase, SOD, and GPx (Takaya et al., 2012).

### Mitochondrial oxidative stress in stroke

**Figure 6:**
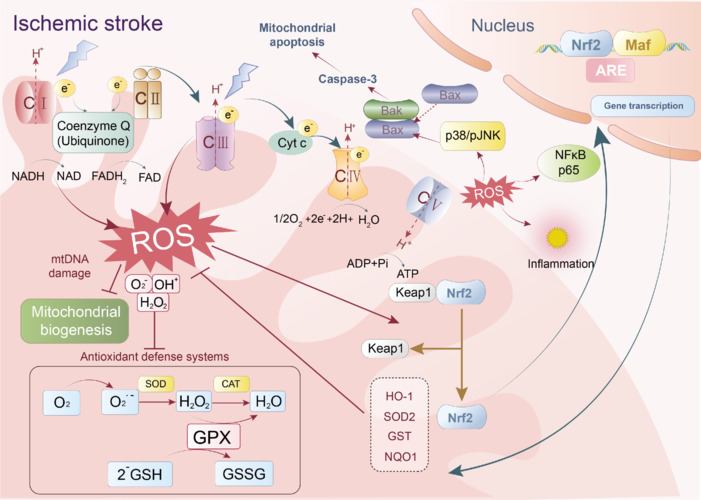
Significant role of mitochondrial oxidative stress in ischemic stroke. Under ischemic conditions, the function of the mitochondrial electron transport chain is impaired, particularly in complex I and complex III. This impairment leads to electron leakage and the production of ROS. Additionally, the activity of intracellular antioxidant enzymes such as SOD, CAT, and GPx decreases, making it difficult to effectively eliminate excessive ROS. The resulting ROS causes damage to mitochondrial DNA and inhibits mitochondrial biogenesis. It also increases the permeability of the mitochondrial outer membrane, triggers the release of pro-apoptotic factors, activates the caspase pathway, and ultimately leads to cell apoptosis. Furthermore, ROS stimulates the production and release of inflammatory factors, initiating inflammatory responses that cause additional damage to surrounding tissues. To counteract these effects, activating the Nrf2 signaling pathway can enhance the expression of antioxidant enzymes, reduce oxidative stress, protect mitochondrial function, and minimize brain damage. Red solid arrows represent ROS-related promotional signaling processes; red T-shaped arrows represent ROS-related inhibitory signaling processes; red dashed arrows represent the transfer of protons (H⁺ ions) along the respiratory chain; brown arrow and dark grey arrow represent NRF2-related signaling processes. ARE: Antioxidant response elements; CAT: catalase; Cyt c: cytochrome c; FAD: flavin adenine dinucleotide; GPX: glutathione peroxidase; GSH: glutathione; GSSG: glutathione disulfide; GST: glutathione transferase; HO-1: heme oxygenase-1; IS: ischemic stroke; Keap1: kelch-like ECH-associated protein 1; Maf: musculoaponeurotic fibrosarcoma; mtDNA: mitochondrial DNA; NAD: nicotinamide adenine dinucleotide; NADH: nicotinamide adenine dinucleotide hydrogen; NQO1: nicotinamide quinone oxidoreductase 1; Nrf2: nuclear factor erythroid 2-related factor 2; ROS: reactive oxygen species; SOD: superoxide dismutase.

One of the core pathological mechanisms of stroke, especially IS, is oxidative stress. Long-term ischemia leads to decreased activities of ETC complexes I and III (Chen et al., 2017), and mitochondrial ROS (mtROS) are produced and maintained at high levels after reperfusion (Li et al., 2012). mtROS accumulation results in the opening of the mPTP and decreased MMP levels, which eventually disrupt the structure and function of mitochondria and rapidly overwhelm antioxidants, especially the critical target for ischemia protection SOD2 (Holley et al., 2011; Manzanero et al., 2013; Teixeira et al., 2018). Active phosphorylated p38 MAPK plays a vital role in Bax translocation to mitochondria under simulated ischemia (Capano and Crompton, 2006). A previous study showed that increased ROS generation can activate the p38/pJNK MAPK/caspase-3 pathway, which ultimately results in mitochondrial apoptosis and disrupts ATP synthesis (Cao et al., 2016). The massive mtROS accumulation can also damage the mtDNA, inhibiting mitochondrial biogenesis (Xia et al., 2020). mtROS is the key stimulus to trigger the activation of the NLRP3 inflammasome through NF-κB to expand the inflammatory response (Cao et al., 2016). Moreover, the damaged mitochondria, in turn, continue to produce ROS to create a vicious cycle of damage in IS (**Figure 6**).

After cerebral hemorrhage, brain tissue is severely damaged, leading to a series of complex pathophysiological changes, of which oxidative stress is one of the key factors. As a result of the severe oxidative stress in the brain after cerebral hemorrhage after ICH, the BBB is disrupted, and inflammatory mediators from the blood, such as thrombin, complement, and fibrin degradation products, enter the brain through the damaged BBB and induce inflammatory reactions in and around the blood clot after HS, and are marked by leukocyte infiltration and microglial cell activation (Chen et al., 2022). Two studies have reported that the duration of neutrophil infiltration peaks within 48 hours and that microglia are maximally activated 7 and 10 days after HS, leading to neuronal damage (Gong et al., 2000; Xue and Del Bigio, 2000). In addition, the hemoglobin contained in the hematoma in the brain plays a major role in regulating oxidative stress after cerebral hemorrhage, releasing large amounts of ROS and disrupting homeostatic balance. Furthermore, oxidative stress following cerebral hemorrhage is closely associated with iron toxicity. After HS, hemoglobin is released and metabolized to Fe^2+^ since it is phagocytosed by microglia and macrophages surrounding the hematoma. This process leads to iron overload and its accumulation in neurons, causing a sudden increase in ROS and disrupting cellular function (Wan et al., 2019).

### Regulation of mitochondrial oxidative stress by natural products

The utilization of antioxidants has been shown to decrease superoxide anion levels in tissues, thereby mitigating the progression of cerebral infarction. Consequently, antioxidant stress is regarded as a promising strategy for alleviating IS injury (Dohare et al., 2014; Shao et al., 2020). TAB, a lanostane-type triterpenoid derived from the *Trametes lactinea* (Berk.) Pat., has demonstrated neuroprotective effects in both *in vitro* and *in vivo* models. Specifically, TAB ameliorates neuronal damage in rats subjected to I/R injury and SH-SY5Y cells exposed to OGD/R. These neuroprotective effects are closely linked to the suppression of miR-10a, which targets PIK3CA, thereby activating the PI3K/Akt/mTOR signaling pathway (Wang et al., 2019). Kukoamine A, a primary bioactive constituent derived from the root bark of *L. chinense* Miller, has been demonstrated to reduce cerebral infarct volume, brain edema, and apoptotic cell count, while enhancing neurological deficit scores. The underlying mechanisms potentially involve the attenuation of brain swelling, modulation of oxidative status, and inactivation of the mitochondrial apoptosis pathway (Liu et al., 2017). Monomethyl lithospermate, a component of *Shenxiong Tongmai* granules, has been shown to significantly enhance the neurological deficit scores in rats subjected to MCAO and to ameliorate the hippocampal neuronal damage induced by I/R. The underlying mechanisms are likely associated with the inhibition of cellular apoptosis via activation of the PI3K/Akt signaling pathway (Yang et al., 2022a). Ginsenoside Rg3, a tetracyclic triterpene diol saponin monomer derived from *Panax notoginseng*, plays a crucial role in mitigating mitochondrial oxidative stress, thereby contributing significantly to the attenuation of cerebral I/R injury. The neuroprotective mechanism of ginsenoside Rg3 is associated with the activation of the Nrf2/HO-1 signaling pathway (Chen et al., 2024a). Danhong injection, a standardized extract derived from *Salvia miltiorrhiza* (*Danshen*) and *Carthamus tinctorius* (*Honghua*), was found to significantly enhance pyruvate dehydrogenase activity by inhibiting the HIF1α/PDK1 signaling pathway. This mechanism effectively mitigates the suppressive impact of I/R on mitochondrial metabolism (**Additional Table 5**).

**Additional Table 5:** Natural products for the treatment of ischemic stroke by targeting mitochondrial oxidative stress

Natural product	Herbal source	Oxidative stress related mode of action	*In vivo/In vitro*	Model	Optimal dose	Treatment effect	Reference
Trametenolic acid B	*Trametes lactinea* (Berk.) Pat.	ROS, SOD, CAT GSH-Px activities and MDA level	*In vivo In vitro*	Rat models of cerebral I/R injury SH-SY5Y cell models of OGD/R injury	20, 40, 80 mg/kg (*in vivo*) 10, 20, 40 μg/mL (*in vitro*)	Trametenolic acid B protects against ODG/R and I/R injury by suppressing miR-10a expression, activating phosphoinositide 3-kinase/AKT/mammalian target of rapamycin signaling pathway, thereby reducing mitochondrial-mediated apoptosis.	Wang et al., 2019
Kukoamine A	*Lycium chinense* (*L. chinense*) Miller	Mn-SOD, Cu/Zn-SOD, MDA, H_2_O_2_	*In vivo*	Rat models of pMCAO	10 mg/kg	Kukoamine A reduces infarct volume both before and after occlusion by decreasing brain swelling, modulating oxidative status, and inactivating the mitochondrial apoptosis pathway.	Liu et al., 2017
Luteolin	*Reseda odorata* L.	Mitochondrial ROS, SOD activity and membrane potential	*In vivo*	Rat models of MCAO/R injury	15, 30,60 mg/kg	Luteolin could upregulate hippocampal SIRT3 in rats suffering from cerebral ischemia, thus activating the downstream AMP-activated protein kinase/mammalian target of rapamycin signaling pathway and promoting the recovery of nerve function.	Liu et al., 2020
Monomethyl lithospermate	*Lithospermum erythrorhizon*	SOD, CAT, GSH, MDA	*In vitro*	SHSY-5Y cell models of OGD/R injury	5, 10, 20 μM	Monomethyl lithospermate has potential protective effects on cells from damage induced by ishcemia/reperfusiion, the possible mechanisms actions are closely related to suppression of cell apoptosis through activating PI3K/Akt signaling pathway.	Yang et al., 2022a
20 (R)-ginsenoside Rg3	*Panax notoginseng*	ROS, MDA, SOD, KEAP1, NRF2, HO-1	*In vivo In vitro*	Rat models of MCAO/ R injury PC12 cell models of OGD/R injury	5, 10, 20 mg/kg 5, 25, or 125 μM	20 (R)-ginsenoside Rg3 attenuates cerebral I/R injury by mitigating mitochondrial oxidative stress via the Nrf2/HO-1 signaling pathway.	Chen et al., 2024a
Methyl protodioscin	Dioscoreaceae	Mul1, SOD2	*In vivo In vitro*	Rat models of I/R injury HT22 cell model of H/ R injury	3 or 10 mg/kg 5 μM	Methyl protodioscin protects rat brain from I/R injury through a mechanism involving regulation of Mul1/SOD2 pathway.	Guo et al., 2019
Monomethyl lithospermate	*Shenxiong Tongmai* granule	SOD, CAT, GSH, MDA	*In vivo In vitro*	Rat models of MCAO/R injury OGD/R-induced SHSY-5Y cells	72.4 μM/kg 5, 10, 20 μM	Monomethyl lithospermate exerts a protective effect against neural damage caused by ischemic stroke *in vivo* and *in vitro* by activating the PI3K/AKT pathway.	Yang et al., 2022a
Sikokianin A	*Wikstroemia indica*	ROS, MDA, SOD	*In vitro*	PC12 cell models of OGD/R injury	0.1, 1, 10 μM	Sikokianin A can activate Nrf2 and downstream heme oxygenase-1 in PC12 cells treated by OGD/R.	Yao et al., 2019
Danhong injection	*Salvia miltiorrhiza* (*Danshen*) and *Carthamus tinctorius* (*Honghua*)	MDA, pyruvate, acetyl CoA, NAD^+^, ATP, SOD, ATP synthase, Na^+^-K^+^-ATPase, T-AOC	*In vivo*	Rat models of I/R injury	0.5, 1.0, 2.0 mL/kg	*Danhong* injection can alleviate cerebral edema after cerebral I/R and protect the ischemic penumbra, with these effects attributed to the regulation of intracellular energy metabolic coupling.	Zeng et al., 2021b

ATP: Adenosine triphosphate; CAT: catalase; GSH: glutathione; I/R: ischemia/reperfusion; MCAO/R: middle cerebral artery occlusion occlusion/reperfusion; MDA: malondialdehyde; OGD/R: oxygen-glucose deprivation/re-oxygenation; pMCAO: permanent middle cerebral artery occlusion; ROS: reactive oxygen species; SOD: superoxide dismutase; T-AOC: total antioxidant capacity.

Celastrol significantly reduces mitochondrial oxidative stress, brain edema, and neuronal apoptosis caused by cerebral hemorrhage by enhancing OPA1-mediated mitochondrial fusion (Diao et al., 2024). Green tea and red tea have been shown to partially prevent motor deficits and striatal oxidative damage induced by HS by reducing lipid peroxidation levels. Specifically, black tea primarily prevents motor deficits, while green tea demonstrates better neuroprotective effects in the early stages (Sosa et al., 2018). *Oleuropein aglycone* significantly reduces the neurological deficits and cerebral edema caused by HS by reducing oxidative stress and maintaining the integrity of the BBB in a dose-dependent manner. Additionally, it inhibits the activation of the MAPK signaling pathway induced by HS. These findings suggest that oleuropein aglycone has potential as a novel therapeutic agent for HS, offering new directions for future clinical applications (Shi et al., 2017). Scutellarin, a flavonoid extracted from the traditional Chinese herb *Erigeron breviscapus*, possesses antioxidant properties and significantly attenuates vasospasm while improving neurological function within 48 hours after SAH by modulating the Erk5-KLF2-eNOS signaling pathway (Li et al., 2016).

## Current Status of Clinical Translation

The number of clinical trial registrations for natural medicines has increased substantially in recent years. Taking TCM as an example, in 2024, the Center for Drug Evaluation (CDE) of the U.S. Food and Drug Administration (FDA) will accept a total of 124 new TCM drugs, of which Class 1.1 innovative TCM drugs account for more than 50%, showing the high enthusiasm for the research and development of innovative TCM drugs and the increase in the speed of review (Miksad and Ryals, 2024). This trend is not only reflected in TCM, but also in the field of natural medicines as a whole, with positive progress in the registration of clinical trials. While natural medicines have made some progress in achieving FDA approval as new drugs, overall, only a few natural medicines have been approved by the FDA. This is mainly due to the complexity and specificity of natural medicines, as well as the strict standards of the FDA for the approval of new drugs.

The complexity of natural medicines and the challenges associated with extraction and separation techniques pose difficulties for patent protection in this field. Despite these challenges, the number of patent applications in the field of natural medicines has been increasing each year recently and the patent layout and protection have also been strengthened. The National Institutes of Health (NIH), as an internationally renowned biomedical research institution, has relatively few funding projects for natural medicines. This is mainly due to the fact that the NIH mainly focuses on research in the field of biomedicine, while natural medicines, as an important part of traditional medicine, make up a relatively small proportion of the NIH’s funding system. However, with the deepening of natural medicine research and the acceleration of the internationalization process, the performance of natural medicines in terms of FDA funding for new drug approval and clinical translational projects is expected to improve.

## Safety Assessment

Natural medicines generally cause fewer adverse reactions and side effects than chemical and synthetic drugs in mainstream medicine. Despite these advantages, the safety of natural medicines in the treatment of serious conditions such as stroke needs to be rigorously evaluated. This is mainly because different individuals may respond differently to medications, and some natural medicines may contain potentially toxic ingredients. Therefore, patients receiving natural medicines for stroke treatment should be closely monitored for the appearance of adverse and toxic reactions.

Safety assessment is a critical step in ensuring the safety of natural medicines for stroke treatment. This step typically includes a series of experimental and clinical studies to evaluate the efficacy, safety, and tolerability of the drug. For example, animal experiments can provide an initial assessment of the toxicity and efficacy of a drug, followed by clinical trials to further verify its safety and efficacy in humans. Additionally, the safe use history is another method for assessing the safety of natural medicines. This approach evaluates the safety of a substance or extract on the basis of its long-term history of use. A natural medicine can be considered to have a high safety profile if it has been widely used throughout history without serious adverse effects.

## Limitations

The use of natural products is associated with certain limitations. Currently, the clinical efficacy data for the use of natural products for stroke treatment are predominantly derived from studies conducted in Asia, resulting in a paucity of efficacy data from other regions. This regional disparity poses challenges in extrapolating the findings from animal models to human populations across diverse geographical areas. This factor has limited the scope to conclusively demonstrate the efficacy of natural products for stroke on a global scale. Furthermore, animal studies do not accurately replicate the toxicity profiles of natural products in humans.

## Conclusions and Future Perspectives

Mitochondria, which are closely linked to stroke, have attracted increasing attention due to their important pathogenic role in metabolic-associated brain diseases. Recent research has elucidated several mechanisms underlying mitochondrial dysfunction, including abnormalities in the respiratory chain and bioenergetics, impaired mitochondrial quality control, dysregulated apoptosis, excessive autophagy, oxidative stress, alterations in mitochondrial transport, and reduced mitochondrial membrane potential within mitochondrial pathways.

Further investigation into these mechanisms may facilitate the development of pharmacological interventions targeting mitochondrial dysfunction in metabolic-associated neurological disorders. Nevertheless, this process is protracted. This paper provides a comprehensive overview of natural products that have been recently examined for their potential to ameliorate metabolic-associated brain diseases. It specifically focuses on the prospective roles of these products as therapeutic agents for stroke, primarily through the mitigation of mitochondrial dysfunction.

Natural products exhibit multi-component, multi-target, and multi-level characteristics, making them suitable for treating stroke at various stages (Zhang et al., 2023a). While natural products may lack the thermodynamic advantage of favorable binding properties shown by many chemical agonists, their pharmacological and economic benefits have garnered notable interest from the scientific community. Consequently, natural products can serve as a valuable complementary therapy for addressing mitochondrial dysfunction in metabolic-associated brain diseases.

Comprehensive information regarding the pharmacology, potential toxicity, and adverse reactions of natural products must be made available before their application. Beyond preclinical investigations, clinical trials are essential for the treatment of stroke using natural products. As previously discussed, the use of natural products often presents with two primary challenges: limitations in clinical applicability and potential toxicity, both of which require further research to identify viable solutions. While natural products are generally characterized by their accessibility and low toxicity, their potential for significant therapeutic efficacy is of paramount importance.

## Additional files:

***Additional Table 1:*** *Natural products for the treatment of ischemic stroke by targeting mitochondrial biogenesis.*

***Additional Table 2:*** *Natural products for the treatment of ischemic stroke by targeting mitochondrial fusion and fission.*

***Additional Table 3:*** *Natural products for the treatment of ischemic stroke by targeting mitochondrial autophagy.*

***Additional Table 4:*** *Natural products for the treatment of ischemic stroke by targeting mitochondrial apoptosis.*

***Additional Table 5:*** *Natural products for the treatment of ischemic stroke by targeting mitochondrial oxidative stress.*

## Data Availability

*All relevant data are within the paper and its Additional files*.
